# Perinatal HIV in Europe: Clinical Advances and Psychosocial Challenges—A Scoping Review

**DOI:** 10.3390/tropicalmed11070200

**Published:** 2026-07-16

**Authors:** Helena Lutchman, Cannelle Michel, Audrey Murat-Ringot, Florence Carrouel

**Affiliations:** 1 Université Lyon 1, P2S UR4129, 69008 Lyon, France; helena.lutchman@univ-lyon1.fr (H.L.); cannelle.fogue-djombou@etu.univ-lyon1.fr (C.M.); audrey.ringot@chu-lyon.fr (A.M.-R.); 2 Hospices Civils de Lyon, 69002 Lyon, France

**Keywords:** HIV, pregnancy, maternal health, MTCT, stigma, migration, Europe

## Abstract

Despite significant biomedical advances in the prevention of mother-to-child transmission (MTCT) of HIV, residual transmission and suboptimal maternal outcomes persist across Europe. The challenge is no longer primarily virological but structural: aggregated ART coverage indicators mask important discontinuities across the perinatal care pathway. This scoping review maps evidence on clinical and psychosocial dimensions of the perinatal HIV pathway among adult women living with HIV (WLHIV) in Europe, and identifies key gaps in their integration across the continuum of care. Following PRISMA-ScR guidelines, five databases were searched for studies published between 2010 and 2026. Of 1881 records identified, 109 met inclusion criteria. Findings were synthesized thematically using an inductively developed framework spanning preconception through long-term postpartum outcomes. MTCT rates declined from approximately 1–1.2% in the mid-2000s to below 0.5% in recent data, and vaginal delivery rates increased markedly with sustained viral suppression. However, persistent inequalities remain among younger women, migrants, and those with histories of injecting drug use. Stigma, migration-related vulnerability, mental health difficulties, constrained disclosure, and socioeconomic insecurity shape engagement with care, influencing treatment continuity, retention, and virological outcomes, particularly during the postpartum period. Equitable perinatal HIV outcomes require integrated care models that combine clinical HIV and obstetric services with mental health support, social needs screening, and migration-sensitive services to address the structural conditions shaping care trajectories. A substantial proportion of WLHIV in Europe originate from high-prevalence regions, making perinatal HIV in this context a direct interface between global infectious disease burden and migration health. Addressing care in European settings therefore contributes to broader global health equity goals in HIV elimination. The protocol was pre-registered on Open Science Framework with registration number: 10.17605/OSF.IO/9BZGY.

## 1. Introduction

Significant progress has been made over recent decades in the prevention of mother-to-child transmission (MTCT) of Human Immunodeficiency Virus (HIV). The global scale-up of antiretroviral therapy (ART), combined with early antenatal screening and improvements in obstetric care, have transformed MTCT from a major public health issue into a largely preventable outcome. In 2023, an estimated 1.2 million pregnant women worldwide were living with HIV [1]. Without intervention, the risk of MTCT ranges from 15% to 45%, whereas it can be reduced to less than 5% [2] with timely diagnosis, effective ART, and appropriate clinical management. Today, 84% of women and girls worldwide have access to ART to prevent MTCT [3], representing a considerable biomedical achievement.

Despite these major biomedical advances, the persistence of residual transmission and suboptimal maternal outcomes reveals that the challenge is no longer primarily virological, but systemic. Aggregated indicators of ART coverage and viral suppression tend to mask important discontinuities across the perinatal care pathway, particularly in terms of timely diagnosis, sustained engagement in care, long-term treatment adherence, and maternal health outcomes beyond delivery [4,5]. As a result, the prevention of MTCT increasingly depends not only on the availability of effective treatments, but on the continuity and integration of care across the entire reproductive trajectory.

The perinatal period, spanning preconception, pregnancy, and the postpartum phase, constitutes a complex and dynamic continuum in which clinical processes intersect with behavioral, social, and structural determinants of health. Women living with HIV (WLHIV) must navigate not only medical requirements such as ART adherence and monitoring, but also a complex interplay of health, social, economic, and cultural challenges. These include gender-based violence, inequalities in access to education and economic resources, stigma and discrimination, and barriers to healthcare access [6]. These vulnerabilities may be intensified during the perinatal period, when women must manage both clinical care requirements and psychosocial stressors related to disclosure, reproductive decision-making, and maternal identity [7,8]. These dimensions are interdependent: psychosocial vulnerabilities may directly affect care engagement and adherence, thereby influencing clinical outcomes, while clinical constraints may shape social experiences and life trajectories.

However, the existing literature remains fragmented. Clinical research has predominantly focused on biomedical outcomes such as viral suppression, obstetric complications, and MTCT rates, whereas psychosocial studies have explored stigma, mental health, and social support in relative isolation. This separation limits the understanding of how these dimensions interact in real-world care pathways and constrains the development of integrated approaches to maternal and child health among WLHIV.

This gap is particularly relevant in the European context. Although Europe benefits from high ART coverage and well-established healthcare systems, it is characterized by substantial heterogeneity in screening policies, care pathways, and access to services. Antenatal HIV screening approaches range from universal systematic testing to opt-in models requiring explicit consent, or the absence of national screening programs, a pattern of variation that persists in recent assessments of the region [9,10]. Care pathways for HIV-positive pregnant women similarly vary, with most countries directing women to specialized HIV services, while other countries such as France, Finland, and the United Kingdom rely on multidisciplinary teams or Sexually Transmitted Infection (STI) clinics [9,11,12]. These structural differences contribute to unequal access to diagnosis, treatment, and prevention of MTCT across countries.

In parallel, the epidemiology of HIV in Europe is strongly shaped by migration dynamics. Indeed, in 2024, migrants accounted for 55.7% of all new HIV diagnoses in Europe, and 75% of MTCT cases occurred within migrant populations [13]. Across European settings, WLHIV during pregnancy are predominantly women of sub-Saharan African (SSA) origin or with a migration background [14]. Migration status intersects with socioeconomic vulnerability, administrative barriers, and reduced access to healthcare, thereby shaping both clinical outcomes and psychosocial experiences [15]. Compared with native-born women, migrant women are more likely to be unaware of their HIV status at conception, to receive a late diagnosis during pregnancy, to present with more advanced immunosuppression, to engage later with antenatal care, and not to return for HIV care in the year following pregnancy [16,17,18,19,20]. Perinatal HIV therefore remains an important global public health challenge despite substantial progress in prevention, particularly in the context of increasing international migration and persistent inequalities in access to antenatal care and HIV services. European countries receive migrants from regions with a high burden of HIV, making perinatal HIV prevention and management closely linked to global infectious disease control and cross-border public health strategies.

Taken together, these findings highlight the need for an integrated understanding of the perinatal pathway among WLHIV, encompassing both clinical and psychosocial dimensions. To date, to our knowledge, no scoping review has systematically mapped these dimensions within the European context. The purpose of this review is therefore to map and synthesize the existing evidence on clinical and psychosocial dimensions of the perinatal pathway among WLHIV in Europe and to identify key gaps in their integration across the continuum of care.

## 2. Methods

The review was conducted in accordance with the PRISMA-ScR “Preferred Reporting Items for Systematic Reviews and Meta-Analyses for Scoping Reviews’’ guidelines [21]. A scoping review methodology was selected to systematically map the extent, range, and nature of the available evidence, rather than to assess causal relationships or intervention effects. This approach is particularly appropriate given the conceptual and methodological heterogeneity of the literature addressing both clinical and psychosocial dimensions of the perinatal pathway among WLHIV. The protocol was pre-registered on Open Science Framework with registration number: 10.17605/OSF.IO/9BZGY.

### 2.1. Research Question

The following research question was formulated using the PCC (Population–Concept–Context) framework: What is known about clinical and psychosocial dimensions of the perinatal pathway (Concept) among women living with HIV (Population) in Europe (Context)?

For the purpose of this review, the perinatal pathway was operationally defined as the period extending from preconception, through pregnancy and childbirth, to the first 24 months postpartum. The 24 months’ timeframe ensures inclusion of longitudinal cohort studies and intervention trials evaluating outcomes relevant to HIV care and maternal health, including retention in care, ART adherence, viral suppression and breastfeeding [22].

This review examines the perinatal pathway of WLHIV, from preconception to the postpartum period, from two complementary perspectives: clinical and psychosocial. The clinical perspective encompasses biomedical and care-related outcomes, including ART adherence, virological control, and obstetric outcomes. The psychosocial perspective considers subjective experiences, including perceptions, decision-making processes, stigma and social interactions.

In terms of geographical scope, the review adopted the WHO European Region framework, comprising 53 member states, including several transcontinental countries such as Russia, Turkey, and Ukraine [10]. This broader scope was adopted to capture the full diversity of perinatal HIV care contexts across Europe, including health systems in non-EU/EEA countries with documented epidemiological relevance to perinatal HIV transmission.

### 2.2. Objectives

The objectives of the review were to:

- Map the existing evidence on the clinical and psychosocial dimensions of the perinatal pathway among WLHIV.
- Analyze how the literature addresses clinical and psychosocial dimensions.
- Identify key gaps to inform future research and the development of more comprehensive and integrated approaches to perinatal HIV care.

### 2.3. Selection of Publications

The search was conducted in PubMed, Embase, Cochrane, Web of Science and ScienceDirect on 3 November 2025 and an update was carried out on 7 April 2026 (covering publications from 1 November 2025 to 31 March 2026). PubMed and Embase were selected for their comprehensive indexing of medical and biomedical research, while Cochrane, Web of Science, and ScienceDirect were included to capture multidisciplinary and high-impact journal publications. Databases such as PsycINFO and CINAHL were not included because the review scope was primarily focused on biomedical and clinical health outcomes rather than psychology-, nursing-, or allied health-specific constructs. Consequently, the selected databases were considered sufficient to capture the relevant literature within the defined scope of this review.

The search strategy was developed based on the PCC framework and structured into three main conceptual blocks: (i) women living with HIV, (ii) perinatal period, and (iii) European context. Within each block, MeSH terms were combined with free-text keywords and truncated terms to maximize sensitivity and account for variations in terminology across databases. The publication year filter (2010–2026) was applied manually using each database’s built-in date filter during search execution. The full search strategies for each database are provided in Supplementary Table S1.

The inclusion criteria were as follows: studies published between 2010 and 2026; peer-reviewed articles with full text available; studies conducted in Europe; studies involving adult women living with HIV; and studies addressing pregnancy or maternal health within the perinatal pathway.

The exclusion criteria were studies addressing HIV without specific reference to the perinatal pathway; focusing primarily on co-infections, comorbidities, or other specific pathologies; focusing solely on pharmacological efficacy; non-peer-reviewed articles; editorials, commentaries, letters, opinion pieces, conference abstracts, oral presentations, posters, books, book chapters, guidelines, or recommendation documents, and case reports; focusing exclusively on questionnaire development, validation, translation, or cultural adaptation and clinical trial protocols with no available results. Grey literature was excluded because it often lacks formal peer review and may provide limited methodological detail, making it difficult to assess study quality and risk of bias consistently. Restricting the review to peer-reviewed publications improved the reliability, comparability, and reproducibility of the evidence synthesis while minimizing the inclusion of incomplete or non-validated data. Adolescent pregnancies were excluded because pregnant adolescents living with HIV constitute a distinct population. Restricting the review to adults reduced clinical and methodological heterogeneity, allowing for a more consistent synthesis of maternal and perinatal outcomes across studies.

First, duplicate records were removed. The articles were saved from search results through Zotero 7.0.32 (by Digital Scholar). The online platform Rayyan (provided by Rayyan Systems, Inc., Boston, MA, USA) automatically flagged potential duplicates and one reviewer (HL) checked and removed duplicates. Uncertain cases were further reviewed by a second reviewer (CM) to confirm inclusion or exclusion. Secondly, prior to screening, a calibration exercise was performed to ensure consistency in applying inclusion and exclusion criteria. Thirdly, title and abstract screening was conducted independently by two reviewers (HL and CM) using Rayyan. Fourthly, full-text screening followed the same independent and blinded process. Inter-reviewer disagreements at both stages were resolved through discussion until consensus was reached.

### 2.4. Data Extraction, Summary of Results, and Evaluation of the Quality

A data extraction grid was collaboratively developed and validated by the two reviewers (HL and CM) prior to data collection. The extraction framework was designed to systematically capture both clinical and psychosocial dimensions of the perinatal pathway. The following data were extracted:

- Author.
- Year.
- Country.
- Study objectives.
- Study design and methods.
- Outcome measures (clinical and/or psychosocial).
- Population characteristics and sample size.
- Key findings.

Data extraction was performed independently by the two reviewers (HL and CM) and cross-checked to ensure accuracy and consistency.

The synthesis followed a descriptive and thematic approach consistent with scoping review methodology. An inductive thematic analysis was used to identify recurrent patterns across studies and to organize findings into major domains, including clinical and psychosocial areas. Coding was performed manually (no software used). Initial codes were generated inductively from the extracted data and applied to relevant segments of the included studies. Coding was conducted independently by two reviewers (HL and CM). Any disagreements in coding were resolved through discussion until consensus was reached. Clinical and psychosocial data were coded separately to preserve conceptual distinction. Themes were then developed by grouping related codes, reviewed iteratively, and refined through repeated comparison across studies to ensure internal coherence and external distinctiveness. Final themes were agreed upon by both reviewers through consensus. Results were summarized narratively without quantitative pooling, reflecting the heterogeneity of study designs and outcomes.

### 2.5. Critical Appraisal

Consistent with PRISMA-ScR recommendations, critical appraisal is not mandatory in scoping reviews and was not used to exclude studies. However, the Mixed-Methods Appraisal Tool (MMAT, version 2018) [23] was applied to provide an overview of the methodological diversity and overall quality of the included studies. Quality assessment was conducted independently by two reviewers (HL and CM), and results were reported descriptively to contextualize the strength and limitations of the evidence base, without influencing study inclusion or the synthesis of findings.

## 3. Results

### 3.1. Article Selection Process

**Figure 1:**
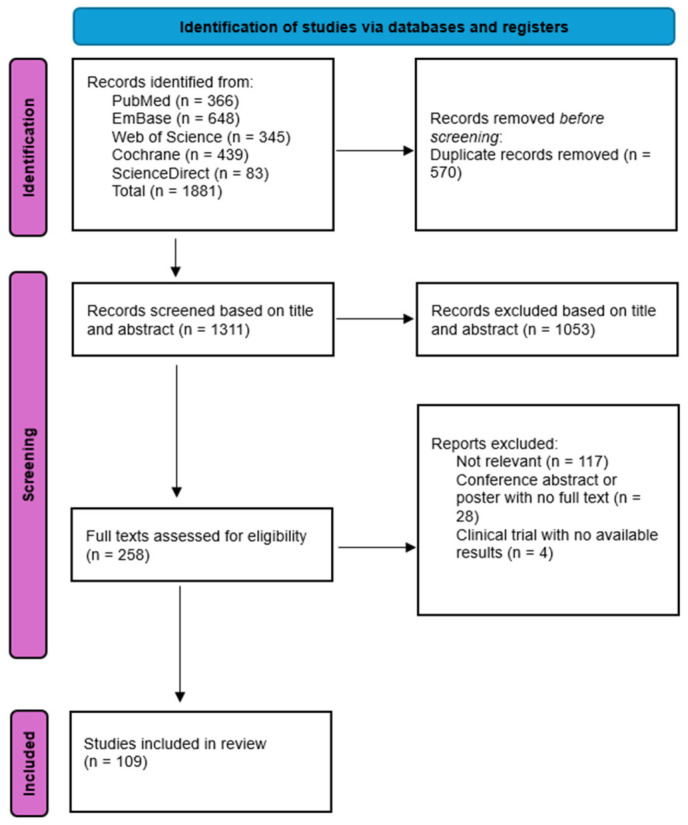
Flowchart of scoping review process (PRISMA-ScR).

Figure 1 summarizes the study selection process. A total of 1881 publications were identified through database searching. After removing duplicates, 1311 publications were screened based on title and abstract.

### 3.2. Characteristics of Included Articles

The characteristics of included studies are presented in Supplementary Table S2. Among the 109 included studies, the evidence was heterogeneous in terms of study design, populations, and outcomes. The review included 109 studies in total, comprising 37 prospective observational cohort studies, 30 retrospective observational cohort studies, 24 cross-sectional studies, 6 longitudinal studies, 3 case–control studies, 6 case series, 1 clinical audit, 1 secondary qualitative data analysis, and 1 decision analytic modelling study.

**Figure 2:**
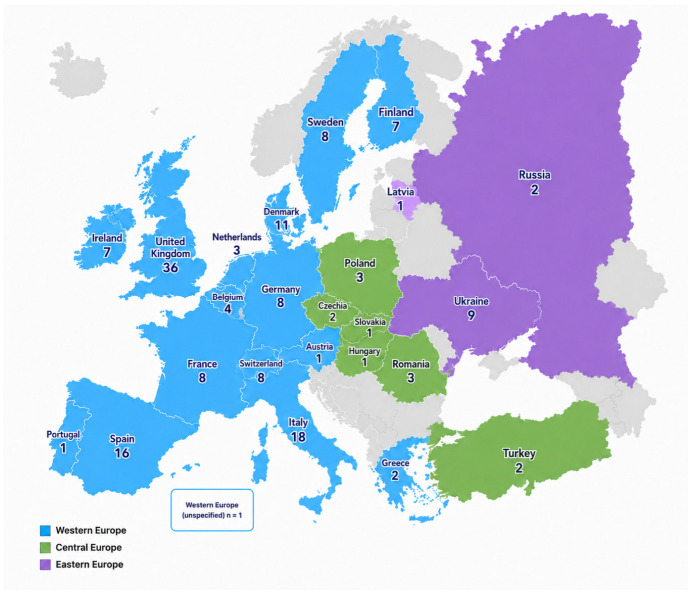
Number of study-country occurrences among included studies.

Studies were conducted across multiple European countries, and several studies included more than one location. Countries were classified into Western, Central, and Eastern Europe according to the WHO European Region grouping [10], under which Western Europe comprised the United Kingdom, Italy, Spain, Denmark, France, Germany, Sweden, Switzerland, Finland, Ireland, Belgium, The Netherlands, Austria, Portugal, and Greece; Central Europe comprised Poland, Romania, Czechia, Hungary, Slovakia, and Turkey; and Eastern Europe comprised Ukraine, Russia, and Latvia. Western European countries were most frequently represented, appearing 139 times in total, while Central and Eastern European countries were each represented considerably less often, appearing 12 times each, as represented in Figure 2 and Table 1 below.

**Figure 3:**
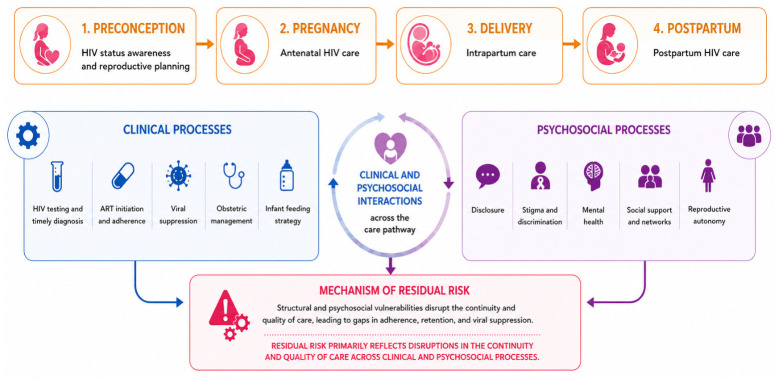
Integrated conceptual model of the perinatal HIV care pathway: interactions between psychosocial and clinical processes.

To structure the synthesis, Table 2 below maps out the occurrence of themes across the review. An integrated conceptual model was further developed based on the synthesis of clinical and psychosocial evidence (Figure 3).

This figure presents an integrated conceptual model of the perinatal HIV care pathway among women living with HIV in Europe, developed from the synthesis of clinical and psychosocial evidence. The clinical processes (including HIV diagnosis, antiretroviral therapy initiation and adherence, virological control, and obstetric management) interact dynamically with psychosocial factors such as stigma, mental health, disclosure, and social support. The model critically highlights how disruptions in care continuity may lead to residual risks.

This analytical framework was used to guide the synthesis of findings presented in the following subsection.

### 3.3. Synthesis of Results

Findings are presented following the analytical framework described above. Results reflect patterns reported across studies rather than causal inference.

#### 3.3.1. Clinical Dimensions

##### Cross-Cutting Factors: Substance Use and Care Engagement

Substance use, particularly injecting drug use (IDU), was reported as a consistent cross-cutting vulnerability operating throughout the perinatal pathway, with prevalence ranging from approximately 15% to 18% across cohorts [24,25].

IDU was associated with advanced HIV disease at presentation, later diagnosis during pregnancy, and lower ART coverage. Studies also reported doubled MTCT risks among women with IDU compared with non-IDU women (10.8% versus 5.9%) [26].

Substance use was also reported in relation to obstetric outcomes including preterm birth, small-for-gestational-age infants, and low birth weight [26]. In the postpartum period, IDU was associated with significantly higher rates of loss to follow-up after delivery, with substance use contributing to disengagement through mechanisms including unstable living conditions, stigma, competing caregiving demands, and poverty [27].

In addition to IDU, tobacco smoking was reported as being associated with fetal growth restriction and low birth weight in multivariable analyses [28].

##### ART Coverage, Adherence and Virological Outcomes

Across cohorts, antenatal ART coverage was high and increased over the time, with an increasing proportion of women receiving ART prior to conception [29,30,31].

Earlier initiation of ART during pregnancy was reported when treatment was not already in place, typically during the first or second trimester. Studies also described increased use of contemporary antiretroviral regimens, including integrase inhibitors [32]. Differences in timing of ART initiation were reported according to patient characteristics, with younger women and those diagnosed during pregnancy more likely to initiate treatment later [33,34]. Where ART was sustained throughout pregnancy and initiated prior to conception, studies reported high rates of viral suppression at delivery and low levels of MTCT [35].

The relationship between ART exposure and preterm birth was inconsistent, with earlier studies reporting associations between combination ART and increased risk of preterm birth, while more recent ones reported declining preterm birth rates over time, particularly in populations with high levels of viral suppression [35,36].

Suboptimal adherence and interruptions in care were reported in relation to residual MTCT events [37]. Postpartum outcomes included reports of virological rebound and treatment interruption among women initiating ART during pregnancy [38,39].

##### Obstetric Outcomes: Mode of Delivery and Preterm Birth

Regarding mode of delivery, studies reported a temporal increase in vaginal delivery rates across multiple cohorts. In pooled European data, vaginal delivery increased from 17% to 52% [40], with similar trends reported in Finland, Spain, and Italy [30,41,42]. Correspondingly, rates of elective caesarean section (CS) declined.

Vaginal delivery was reported in association with suppressed viral load at delivery, whereas caesarean section was reported in cases of detectable viraemia. Earlier cohorts reported higher CS rates, including 88.7% in Switzerland, 74% in the United Kingdom, and 92.3% in Italy [29,43,44].

Comparative studies reported no significant differences in labour or neonatal outcomes between WLHIV and HIV-negative women when clinical criteria for vaginal delivery were met [45]. Registry data confirmed that increasing vaginal delivery was not accompanied by adverse neonatal outcomes or increased MTCT [41,42].

Variation in clinical practice was reported across settings. Elective CS rates remained elevated in some contexts, including among women with suppressed viral load [41,46]. In the most recent cohorts, CS was increasingly driven by obstetric rather than HIV-specific indications [41].

Preterm birth (PTB) rates varied across cohorts and were reported to range from 8% in United Kingdom surveillance data [47] to over 27% in Swiss cohorts [29], with higher rates (36.5%) in earlier studies [48]. Studies reported associations between PTB and markers of HIV disease severity, including lower CD4 counts and detectable viraemia during pregnancy [18,49,50]. Early PTB, defined as less than 32 weeks of gestation, was reported in association with these factors.

Additional factors reported in association with PTB included injecting drug use and social deprivation [25], as well as older maternal age [29,49]. However, this association with maternal age was not consistently reported after adjustment in larger studies [51]. Studies did not consistently report an independent association between ART exposure and PTB risk after adjustment for confounding factors, and no consistent association was reported between specific ART regimens and PTB [49,50].

##### Mother-to-Child Transmission: Declining Rates and Persistent Gaps

MTCT rates declined across the study period. Large European cohorts reported reductions from approximately 1–1.2% in the mid-2000s [46,52] to below 0.5% in more recent data [47]. Transmission rates approaching zero were reported in contexts of undetectable maternal viral load at delivery [37], and elimination of MTCT since 2000 was reported in some hospital-based series [43]. Sustained combination ART was identified as the primary protective factor, with evidence of up to a sevenfold reduction in transmission compared with zidovudine monotherapy [53].

Residual transmission continued to occur across the study period and was reported in association with identifiable risk factors. Late or absent HIV diagnosis during pregnancy, delayed or absent ART initiation, incomplete intrapartum prophylaxis, and detectable viraemia at delivery were identified as contributors to preventable transmission [37,54]. A disproportionate share of transmission events occurred among women without antenatal treatment exposure [55]. Advanced HIV disease and low CD4 counts were also reported as risk factors for transmission [18,56], as were high viral replication in late pregnancy and preterm birth [50]. Acute seroconversion during pregnancy, resulting in undetected infection at delivery, was reported as a contributor to isolated transmission events in some studies [37,54].

Care engagement was reported as a further determinant of MTCT risk across multiple studies. High and sustained ART coverage throughout the reproductive continuum was associated with transmission rates approaching zero, while gaps in adherence, late diagnosis, and treatment interruption were associated with residual transmission [35,52,55]. Declining MTCT trends over time were reported at the population level in Spain, France, and the United Kingdom, in association with improved integration of HIV care into antenatal services [30,35,37].

##### Postpartum Retention in Care and Loss to Follow-Up

Postpartum engagement in HIV care was a point of attrition, despite generally high antenatal ART coverage. Stable preconception ART use and longer treatment duration were associated with improved retention and reduced virological failure [57]. Some evidence indicated improved care engagement during pregnancy and the immediate postpartum period compared with pre-pregnancy stages, although this effect was not consistently sustained over time [58].

Postpartum disengagement was reported in association with a range of structural and psychosocial factors. Rates of loss to follow-up (LTFU) and delayed postnatal clinic attendance ranged from 12% to 34% in some cohorts. History of IDU, non-suppressed viral load at delivery, younger age, and socioeconomic disadvantage were reported as determinants of higher LTFU risk [20,24,59]. Structural barriers associated with disengagement included poverty, travel distance, administrative requirements, and difficulties accessing ART prescriptions after delivery [27]. Psychosocial factors reported in association with disengagement included HIV-related stigma, misinformation, AIDS denialism, reduced social support, and competing demands of infant care [27]. Depression was linked with poorer infant follow-up, and illicit drug use was associated with maternal virological failure in the postpartum period [5].

#### 3.3.2. Psychosocial Dimensions

##### Disclosure, Perceived Support, and Stigma Experiences

HIV-related stigma and social support modulated women’s experiences across the perinatal pathway. Stigma was associated with constrained disclosure, reduced access to support, and lower engagement with healthcare services, while positive support from partners and peers was associated with improved wellbeing and adjustment [60].

Disclosure patterns varied across studies. The majority of women disclosed their HIV status to at least one person, including a partner (90%) or a parent (58%) [61], while 10% had not disclosed to their partner and 5% had not disclosed to anyone. Limiting disclosure to a small trusted circle was reported as a response to fear of social isolation [62,63], and was associated with reduced access to support [64].

Within healthcare settings, positive experiences were associated with perceived provider empathy, continuity of care, and multidisciplinary approaches addressing both clinical and psychosocial needs [65,66]. Interdisciplinary collaboration between obstetricians and infectious disease specialists was identified as a feature of effective perinatal care [67]. Negative experiences were also reported, including breaches of confidentiality, inconsistent communication, and perceived discrimination by healthcare professionals [68]. Internalized stigma, including feelings of guilt and moral responsibility related to HIV transmission risk in the context of sexuality and childbearing, was reported as a constraint on reproductive autonomy and a contributor to care avoidance [69].

Infant feeding was identified as a specific domain of stigma-related concern. The inability to breastfeed in accordance with social and cultural expectations was reported as a source of anxiety regarding inadvertent HIV disclosure and social judgement from family and community members [69].

##### Psychological Distress and Unmet Mental Health Needs

Depressive symptoms were reported in approximately one quarter to one third of women across the perinatal period, with prevalence ranging from 27% antenatally to 36% at three months postpartum [66,70]. Higher rates were consistently reported in the postnatal phase. Psychological stress during pregnancy was reported by the majority of women in one cohort, and distress specifically related to the inability to breastfeed was reported by 72% of participants [71].

Social factors were more consistently identified as risk factors for poor mental health than clinical factors. Living alone, single parenthood, and low self-efficacy in managing ART or infant care were associated with higher rates of depressive symptoms [70]. Loneliness and low social support were reported as independent predictors of both antenatal and postnatal depression, with WLHIV reporting significantly lower social support than HIV-negative women [66]. In cohorts with detailed psychosocial data, the majority of pregnancies were accompanied by concurrent psychological or social problems, including depression, partner-related concerns, financial difficulties, housing instability, and childcare challenges [72].

Initial reactions to HIV diagnosis including shock, fear, thoughts of self-harm, and reconsideration of the pregnancy were reported across qualitative studies [64]. Adjustment over time was reported in several studies, though this process was described as variable and influenced by ongoing stigma [69,73]. Pregnancy was reported by some women as restorative of a sense of normality, while fear of MTCT, reported by 43.5% of women in one cohort, was associated with avoidance of infant contact in 40% of cases, with reported consequences for mother–infant bonding [71].

Access to psychological support was reported as limited relative to need. Unmet need was greatest in the postpartum period, with a substantial proportion of women who sought support not accessing it, despite higher service contact rates during the antenatal period [70].

##### Reproductive Intentions, Contraception, and Pregnancy Outcomes

Reproductive desire was reported in approximately half of WLHIV across studies [74,75]. HIV diagnosis was reported as a source of disruption to reproductive self-perception, with associated ambivalence affecting both conception planning and contraceptive behavior in some studies [73].

A gap between reproductive intentions and contraceptive practice was reported across multiple studies. Contraceptive use was frequently inconsistent, with condoms reported as the predominant method and long-acting reversible contraception remaining underutilized [76,77]. No contraceptive use despite sexual activity was reported by 35.5% of women in one cohort [76], and unintended pregnancies were reported among women using condoms or oral hormonal methods.

Planned conception under clinical supervision in serodiscordant couples was reported as safe and effective when viral suppression was achieved, with high pregnancy success rates and low transmission risk [78,79]. Factors associated with reproductive success included younger maternal age and absence of fertility disorders. Outcomes of assisted reproductive technologies were reported as less favorable in couples where the woman was HIV-positive, including lower cumulative pregnancy and live birth rates and greater treatment burden compared with HIV-negative women [80].

Induced abortion rates were reported as higher following HIV diagnosis in earlier studies, in association with fear of MTCT, socioeconomic disadvantage, and IDU, and decreased over time in parallel with improvements in clinical management [81,82]. In more recent studies, HIV status was not identified as an independent predictor of abortion after adjustment for reproductive and social factors, with pregnancy intendedness, relationship context, and reproductive history reported as stronger predictors [83].

##### Infant Feeding Decisions

Infant feeding decisions were consistently reported as a source of psychosocial complexity in the postpartum period. The inability to breastfeed was reported as among the most distressing aspects of the perinatal experience for WLHIV, associated with perceived threats to cultural identity and maternal self-perception [59,71]. In settings where replacement feeding is recommended, formula feeding was reported as carrying social risk: the majority of women reported being questioned by friends or family about their feeding choice, and fear that formula feeding would inadvertently disclose HIV status was commonly reported [63,84]. Replacement feeding was reported as a perceived departure from cultural expectations of motherhood in several studies [85,86].

Inconsistent and evolving clinical guidance was reported as a determinant of feeding decision-making. Confusion about the compatibility of breastfeeding with viral suppression in the context of Undetectable = Untransmittable (U = U) was reported, and infant feeding discussions were described as largely provider-initiated rather than woman-led [87]. Emotional responses to not breastfeeding, including grief, guilt, and the need to justify the decision, were reported across qualitative studies, with cultural expectations identified as shaping both the experience and coping strategies employed [88]. Father involvement in feeding decisions was reported as variable, influenced by his awareness of the woman’s HIV status, relationship quality, and confidence in the feeding decision, with single women more frequently reported as navigating these decisions without paternal involvement [89].

In cohorts where breastfeeding occurred within a framework of suppressed viral load and close clinical monitoring, no cases of postnatal transmission were documented [90,91]. An increasing trend of breastfeeding among WLHIV over time was reported in some settings [92]. Early cessation of breastfeeding among women who had intended to breastfeed was reported in association with inadequate lactation support, unclear guidance, and practical difficulties [85], indicating that rising uptake has not been matched by adequate clinical infrastructure to sustain it. These findings suggest that sustained breastfeeding may depend not only on clinical eligibility but also on consistent, provider-led counselling and structured lactation support throughout the postpartum period. Strengthening postnatal support systems and ensuring clear, harmonized infant feeding guidance may therefore be critical to aligning intended and actual feeding practices among women living with HIV.

##### Quality of Included Articles

The full quality assessment of included studies is presented in Supplementary Tables S3 and S4. Across all design types, research questions (S1) and data appropriateness (S2) received predominantly positive ratings, with “Yes” assigned in 67–100% of studies depending on design. Qualitative studies (n = 15) were generally of high methodological quality. Data collection and study approach were consistently appropriate across all studies (100%). Findings were adequately derived from data in 14 studies (93%), and interpretation was well supported in the same proportion. The main limitation concerned clarity in research question formulation, with 5 studies (33%) rated as unclear (“Can’t tell”) on S1–S2. Minor concerns were identified for coherence (2 studies, 13%) and substantiation of interpretation (1 study, 7%).

Quantitative non-randomized studies (n = 82) demonstrated strong measurement validity (98% appropriate), but notable weaknesses in internal validity. Participant representativeness was inconsistent, with only 40 studies (49%) rated as adequate and 42 (51%) rated as unclear or inadequate. Outcome data completeness was a key limitation, with only 16 studies (20%) meeting criteria and 26 (32%) rated inadequate. Consideration of confounding showed mixed performance (55% adequate), while fidelity of intervention or exposure was often insufficiently reported (61% adequate; 38% unclear). Overall, internal validity domains showed greater variability than measurement-related domains.

Quantitative descriptive studies (n = 6) were generally robust across most quality criteria. Sampling strategies were appropriate in all studies (100%), and most studies also demonstrated adequate measurement and statistical analysis (83% each). However, representativeness of samples was more variable (50% adequate), and risk of nonresponse bias showed no consistent pattern, with ratings evenly distributed across categories (33% each).

Mixed-methods studies (n = 4) showed the greatest variability in quality. All studies met screening criteria (100%). However, justification for mixed-methods design was limited (25% adequate; 50% inadequate). Integration of methods and interpretation of integrated findings were inconsistent (both: 50% adequate; 25% inadequate). The two more recent mixed-methods studies performed considerably better across all criteria. Given the small number of mixed-methods studies included, broader conclusions about this design type should be drawn with caution.

## 4. Discussion

This review highlights both the considerable clinical progress achieved in perinatal HIV care across Europe and the structural and psychosocial conditions that continue to shape whether this progress translates into equitable outcomes.

The evidence synthesized in this review points to a central interpretive claim: under conditions of sustained virological control and uninterrupted care engagement, MTCT has become largely preventable in high-resource settings. This reframes residual transmission as associated with a disruption of care continuity rather than a limitation of available biomedical tools. Late diagnosis and delayed ART initiation remain concentrated among younger and socially disadvantaged women, meaning the biomedical success of the past decade has not been evenly distributed; the populations for whom transmission risk remains highest are precisely those least reached by antenatal engagement [33,34,55]. Guideline inconsistency, particularly around infant feeding and delivery mode, compounds this unevenness by leaving implementation to institutional discretion rather than structured protocol [41,87]. Closing this gap will require investment not only in clinical pathways but in preconception planning, psychosocial support, and continuity across the reproductive trajectory—domains that fall outside the scope of biomedical guidelines as currently structured.

A second, related claim emerging from this synthesis is that psychosocial and clinical dimensions of perinatal HIV care are not parallel tracks but mutually constitutive. Stigma, mental health, and social vulnerability do not simply co-occur with poor clinical outcomes; the reviewed evidence suggests they actively mediate them, operating as proximal drivers of adherence, retention, and viral suppression rather than background correlates [60,72,93]. This has a practical implication that the current literature has not adequately addressed: framing non-adherence or disengagement primarily as behavioral or motivational problems risks misidentifying both the mechanism and the point of intervention. Non-disclosure to partners, for instance, is better understood as a response to perceived social risk than an isolated behavioral choice [94], and interventions aimed at improving disclosure rates without addressing the underlying risk perception are unlikely to succeed.

The postpartum period illustrates this interdependence most sharply and represents the clearest evidence gap identified by this review. It is the point at which clinical risk (viral rebound following pregnancy-initiated ART) and psychosocial risk (isolation, competing infant-care demands, unresolved stigma) converge most acutely, yet it is also the point at which care models are structurally weakest, typically front-loading resources into the antenatal period and tapering support just as vulnerability [27,38].

### 4.1. Implications for Practice and Future Research

The evidence indicates that psychosocial vulnerability is a central determinant of clinical outcomes in perinatal HIV care, not a secondary consideration. Care models that focus primarily on biomedical management are unlikely to achieve equitable outcomes, particularly for women who are socially marginalized, diagnosed late, or insufficiently supported across the reproductive continuum. Several specific implications follow from the evidence synthesized here.

Postpartum care requires particular attention. The evidence consistently identifies the period immediately after delivery as a point of concentrated risk: loss to follow-up is highest, virological rebound risk is elevated for women who initiated ART during pregnancy, and mental health need peaks while access to support contracts. The women most at risk of postpartum disengagement have a largely identifiable profile (younger age, history of injecting drug use, pregnancy-initiated ART, low viral suppression at delivery, social isolation, and poverty), and this profile should inform the targeting of intensified postpartum support. Generic recommendations for continuity of care are insufficient; the evidence supports the development of structured transition protocols at the point of delivery that anticipate rather than reactively manage disengagement.

Infant feeding counselling requires specific reform. The finding that feeding discussions are largely provider-initiated rather than woman-led, combined with documented confusion about U = U and its implications for breastfeeding, points to a specific deficit in shared decision-making practice. Clinical guidance has advanced more rapidly than the communication frameworks through which it is delivered to women. Counselling approaches should be structured around individual women’s circumstances, cultural contexts, and relational environments, rather than around the delivery of standardized recommendations. Father and partner involvement, where relevant and safe, should be actively incorporated given its reported influence on feeding decisions.

For future research, more consistent operationalization of psychosocial constructs, including stigma, social support, and psychological distress, is needed to improve comparability across studies and support the development of mechanism-based interventions. Future primary research should also be explicitly designed to address the geographic and methodological gaps identified here, with deliberate investment in qualitative and mixed-methods research in Eastern European settings and among migrant populations, conducted in partnership with researchers embedded in those contexts. Furthermore, the use of longitudinal study designs could clarify causal relationships between psychosocial factors and clinical outcomes.

### 4.2. Strengths and Limitations of This Review

This review has several strengths. To our knowledge, it represents the first scoping review to systematically map the evidence on the perinatal HIV pathway in the European context. Methodological rigor was ensured through adherence to PRISMA-ScR guidelines, as detailed in Supplementary Table S5, and the involvement of two independent reviewers throughout the screening and data extraction process, limiting the risk of selection bias. The inclusion of 109 studies across multiple European countries enabled a broad mapping of the evidence landscape and facilitated the identification of recurring patterns across heterogeneous settings.

Several limitations should nevertheless be acknowledged. A first limitation is the exclusion of pregnant adolescents living with HIV. Adolescents constitute a distinct population with specific biological, psychosocial, and healthcare needs, and their maternal and perinatal outcomes may differ from those of adult women. Consequently, the findings of this review cannot be generalized to adolescent pregnancies. Secondly, studies for which no full-text version was available were excluded. Although this affected only a limited number of records, potentially relevant evidence indexed in bibliographic databases but inaccessible through institutional subscriptions or open-access sources may have been missed. Thirdly, grey literature, including policy documents, governmental reports, clinical guidelines, conference proceedings, and other non-peer-reviewed sources, was not included. This decision was made to ensure methodological consistency and to limit the inclusion of studies lacking sufficient methodological detail. However, this approach may have excluded practice-based evidence and recent policy developments that have not yet been published in peer-reviewed journals. Furthermore, reliance on peer-reviewed publications may have introduced publication bias, as studies reporting statistically significant or positive findings are generally more likely to be published than studies with null or negative results. Another limitation relates to the composition of the available evidence base. Quantitative observational studies predominated, whereas qualitative and mixed-methods research remained comparatively limited. As a consequence, experiential and relational dimensions of the perinatal HIV pathway, including stigma, disclosure, and lived experiences of care, are less extensively documented than biomedical outcomes. Finally, substantial heterogeneity in study designs, populations, outcome definitions, and methodological quality limited direct comparability across studies and precluded quantitative synthesis. Although study quality was assessed using the MMAT, variability in participant representativeness, completeness of outcome data, and adjustment for potential confounding factors should be considered when interpreting the findings. Overall, these limitations should be taken into account when interpreting the results. Nevertheless, they do not diminish the value of this review as a comprehensive mapping of the current evidence base, and they highlight priorities for future research, particularly the need for more standardized reporting, greater representation of under-studied populations, and increased integration of clinical and psychosocial dimensions in perinatal HIV research.

## 5. Conclusions

This review has mapped the clinical and psychosocial dimensions of the perinatal HIV pathway across documented European contexts, demonstrating that while ART coverage, viral suppression, and MTCT prevention have improved considerably, gains remain unevenly distributed among socially marginalized, late-diagnosed, or inadequately supported women. A central finding is that clinical and psychosocial factors are not independent: mental health, stigma, disclosure, and structural disadvantage are closely associated with adherence, care engagement, and virological outcomes. Care models that address biomedical needs without systematic attention to these factors are unlikely to achieve equitable outcomes, particularly at high-risk transition points such as diagnosis during pregnancy and the postpartum period. This review therefore supports integrating mental health assessment, social needs screening, and individualized counselling into routine perinatal HIV care in the settings examined. Significant evidence gaps remain, including inconsistent measurement of psychosocial constructs, and limited data from Eastern Europe, which bears a disproportionate share of HIV burden and yet remains underrepresented in the accessible literature. These areas require methodologically rigorous, theoretically informed research to develop care models that respond to the full complexity of the perinatal HIV pathway across the broader European region.

## Figures and Tables

**Table 1:** Country representation across the included studies. Records correspond to the number of countries studied in the included articles.

West		Center		East	
Country	Records	Country	Records	Country	Records
United Kingdom	36	Poland	3	Ukraine	9
Italy	18	Romania	3	Russia	2
Spain	16	Czechia	2	Latvia	1
Denmark	11	Hungary	1		
France	8	Slovakia	1		
Germany	8	Turkey	2		
Sweden	8				
Switzerland	8				
Finland	7				
Ireland	7				
Belgium	4				
The Netherlands	3				
Greece	2				
Austria	1				
Portugal	1				
Western Europe (unspecified)	1				
Total	139		12		12

**Table 2:** Evidence synthesis by theme.

Theme and Perinatal Stage	Studies (n)	Main Finding/Evidence Gap
ART: Preconception—Postpartum	46	ART coverage > 90% in recent cohorts. Earlier initiation, more integrase inhibitors over time. High suppression + early ART → low MTCT. Adherence lapses linked to residual MTCT, viral rebound postpartum. **Gap:** Inconsistent ART–preterm birth association; no pregnancy/postpartum adherence interventions.
Mother-to-Child Transmission: Pregnancy– Delivery	34	MTCT down from ~1–1.2% to <0.5%. Near-zero with undetectable VL at delivery. Residual cases: late diagnosis, late ART, detectable VL. **Gap:** No standardized cross-country MTCT comparison.
Obstetric outcomes: Delivery	30	Vaginal delivery rising and CS declining. No rise in adverse outcomes or MTCT. PTB rates vary widely. PTB linked to viraemia, low CD4, IDU, deprivation. **Gap:** Patient delivery preference rarely reported; ART regimen–PTB link unresolved.
Disclosure, support, and stigma: Preconception—Postpartum	23	90% disclose to partner. 5–10% disclose to no one. Continuity of care, provider empathy → better experience. Confidentiality breaches, discrimination, internalized stigma reported. **Gap:** No disclosure-support interventions; no cross-country patient-experience measures.
Reproductive intentions: Preconception	17	~50% report reproductive desire. Condoms dominant, LARC underused. Unintended pregnancy common. Supervised conception in serodiscordant couples safe & effective. Abortion rates declining. HIV no longer independent predictor. **Gap:** Preconception counselling uptake unmeasured; No LARC uptake studies
Infant feeding: Postpartum	14	Inability to breastfeed: major distress, identity threat. Fear of disclosure via formula feeding. Guidance inconsistent. U = U confusion. No transmissions when breastfeeding under suppression + monitoring. **Gap:** Safety data limited to small single-country cohorts; No culturally tailored feeding support evaluated.
Postpartum retention in care: Postpartum	8	LTFU/delayed attendance risk factors: IDU, unsuppressed VL, younger age, deprivation. Structural + psychosocial barriers to engagement. **Gap:** No postpartum retention interventions evaluated.
Substance use: Preconception—Postpartum	7	IDU: later diagnosis, lower ART coverage. MTCT risk ~2× higher with IDU (10.8% vs. 5.9%). Linked to LTFU, preterm birth, LBW. Smoking linked to FGR, LBW. **Gap:** No perinatal substance-use intervention studies.
Psychological distress: Preconception—Postpartum	5	Depression: ~25–36% (peak postpartum). Social factors > clinical factors as predictors. Unmet mental health need, esp. postpartum. **Gap:** No perinatal mental health interventions; longitudinal trajectories unclear.

Note. n = unique included studies mapped to each theme; studies may contribute to more than one theme. ART = antiretroviral therapy; cART = combination antiretroviral therapy; CS = caesarean section; FGR = fetal growth restriction; HEU = HIV-exposed uninfected; HIV = human immunodeficiency virus; IDU = injecting drug use; LARC = long-acting reversible contraception; LBW = low birthweight; LTFU = loss to follow-up; MTCT = mother-to-child transmission; PTB = preterm birth; U = U = Undetectable = Untransmittable; VL = viral load; WLHIV = women living with HIV.

## Data Availability

No new data were created or analyzed in this study. Data sharing is not applicable to this article.

## Supplementary Materials

The following supporting information can be downloaded at: https://www.mdpi.com/article/10.3390/tropicalmed11070200/s1, Supplementary Table S1. Search equations for each database. Supplementary Table S2. Data charting and extraction of included studies. Supplementary Table S3. Mixed-Methods Appraisal Tool Quality Assessment. Supplementary Table S4. MMAT Quality Assessment by Study Design and Criterion. Supplementary Table S5. Preferred Reporting Items for Systematic reviews and Meta-Analyses extension for Scoping Reviews (PRISMA-ScR) Checklist.
