# Supplementary material for: Perinatal HIV in Europe: Clinical Advances and Psychosocial Challenges—A Scoping Review
Source: Trop Med Infect Dis. 2026 Jul 16;11(7):200. doi: 10.3390/tropicalmed11070200 (PMC13416566; doi:10.3390/tropicalmed11070200)
Supplement: Supplementary file 1 [file tropicalmed-11-00200-s001.zip › tropicalmed-4397534-supplementary.pdf]

Supplementary Table S1: Search equations for each database

The publication year filter (2010–2026) was applied manually using each database's built-in date filter during search execution.

Database	Research Equation
PubMed	#1. "hiv"[MeSH Terms] OR "hiv infections"[MeSH Terms] OR "human immunodeficiency virus*"[Title/Abstract] OR "hiv"[Title/Abstract]<br><br>#2. "pregnancy"[MeSH Terms] OR "pregnant people"[MeSH Terms] OR "pregnan*"[Title/Abstract] OR "child bearing"[Title/Abstract] OR "childbearing"[Title/Abstract] OR "mothers"[MeSH Terms] OR "maternal health"[MeSH Terms] OR "mother*"[Title/Abstract]<br><br>#3. "CD4 Lymphocyte Count"[MeSH Terms] OR "Viral Load"[MeSH Terms] OR "Medication Adherence"[MeSH Terms] OR "Patient Compliance"[MeSH Terms] OR "Anti-Retroviral Agents"[MeSH Terms] OR "infectious disease transmission, vertical"[MeSH Terms] OR "drug resistance, viral"[MeSH Terms] OR "CD4"[Title/Abstract] OR "Viral Load"[Title/Abstract] OR "HIV RNA"[Title/Abstract] OR "adherence"[Title/Abstract] OR "compliance"[Title/Abstract] OR "ART"[Title/Abstract] OR "ARV"[Title/Abstract] OR "vertical transmission"[Title/Abstract] OR "MTCT"[Title/Abstract]<br><br>#4. "Social Support"[MeSH Terms] OR "Mental Health"[MeSH Terms] OR "Quality of Life"[MeSH Terms] OR "Depression"[MeSH Terms] OR "Anxiety"[MeSH Terms] OR "Parenting"[MeSH Terms] OR "Family"[MeSH Terms] OR "stress, psychological"[MeSH Terms] OR "Social Isolation"[MeSH Terms] OR "Social Support"[Title/Abstract] OR "stigma"[Title/Abstract] OR "Mental Health"[Title/Abstract] OR "Quality of Life"[Title/Abstract] OR "Depression"[Title/Abstract] OR "Anxiety"[Title/Abstract] OR "coping"[Title/Abstract] OR "Parenting"[Title/Abstract] OR "disclosure"[Title/Abstract] OR "Family"[Title/Abstract] OR "psychological stress"[Title/Abstract] OR "Social Isolation"[Title/Abstract]<br><br>#5. "europe"[MeSH Terms] OR "Albania"[MeSH Terms] OR "Andorra"[MeSH Terms] OR "Armenia"[MeSH Terms] OR "Austria"[MeSH Terms] OR "Azerbaijan"[MeSH Terms] OR "republic of belarus"[MeSH Terms] OR "Belgium"[MeSH Terms] OR "bosnia and herzegovina"[MeSH Terms] OR "Bulgaria"[MeSH Terms] OR "Croatia"[MeSH Terms] OR "Cyprus"[MeSH Terms] OR "czech republic"[MeSH Terms] OR "Denmark"[MeSH Terms] OR "Estonia"[MeSH Terms] OR "Finland"[MeSH Terms] OR "France"[MeSH

	Terms] OR ("Georgia"[MeSH Terms] OR "georgia republic"[MeSH Terms]) OR "Germany"[MeSH Terms] OR "Greece"[MeSH Terms] OR "Hungary"[MeSH Terms] OR "Iceland"[MeSH Terms] OR "Ireland"[MeSH Terms] OR "Italy"[MeSH Terms] OR "Kazakhstan"[MeSH Terms] OR "Latvia"[MeSH Terms] OR "Lithuania"[MeSH Terms] OR "Luxembourg"[MeSH Terms] OR "Malta"[MeSH Terms] OR "Moldova"[MeSH Terms] OR "Monaco"[MeSH Terms] OR "Montenegro"[MeSH Terms] OR "Netherlands"[MeSH Terms] OR "republic of north macedonia"[MeSH Terms] OR "Norway"[MeSH Terms] OR "Poland"[MeSH Terms] OR "Portugal"[MeSH Terms] OR "Romania"[MeSH Terms] OR "Russia"[MeSH Terms] OR "san marino"[MeSH Terms] OR "Serbia"[MeSH Terms] OR "Slovakia"[MeSH Terms] OR "Slovenia"[MeSH Terms] OR "Spain"[MeSH Terms] OR "Sweden"[MeSH Terms] OR "Switzerland"[MeSH Terms] OR "Turkey"[MeSH Terms] OR "Ukraine"[MeSH Terms] OR "united kingdom"[MeSH Terms] OR "european people"[MeSH Terms] OR "europ*"[Title/Abstract] OR "Albania"[Title/Abstract] OR "Andorra"[Title/Abstract] OR "Armenia"[Title/Abstract] OR "Austria"[Title/Abstract] OR "Azerbaijan"[Title/Abstract] OR "Belarus"[Title/Abstract] OR "Belgium"[Title/Abstract] OR ("Bosnia"[Title/Abstract] AND "Herzegovina"[Title/Abstract]) OR "Bulgaria"[Title/Abstract] OR "Croatia"[Title/Abstract] OR "Cyprus"[Title/Abstract] OR "czech republic"[Title/Abstract] OR "Denmark"[Title/Abstract] OR "Estonia"[Title/Abstract] OR "Finland"[Title/Abstract] OR "France"[Title/Abstract] OR "Georgia"[Title/Abstract] OR "Germany"[Title/Abstract] OR "Greece"[Title/Abstract] OR "Hungary"[Title/Abstract] OR "Iceland"[Title/Abstract] OR "Ireland"[Title/Abstract] OR "Italy"[Title/Abstract] OR "Kazakhstan"[Title/Abstract] OR "Latvia"[Title/Abstract] OR "Lithuania"[Title/Abstract] OR "Luxembourg"[Title/Abstract] OR "Malta"[Title/Abstract] OR "Moldova"[Title/Abstract] OR "Monaco"[Title/Abstract] OR "Montenegro"[Title/Abstract] OR "Netherlands"[Title/Abstract] OR "north macedonia"[Title/Abstract] OR "Norway"[Title/Abstract] OR "Poland"[Title/Abstract] OR "Portugal"[Title/Abstract] OR "Romania"[Title/Abstract] OR "Russia"[Title/Abstract] OR "san marino"[Title/Abstract] OR "Serbia"[Title/Abstract] OR "Slovakia"[Title/Abstract] OR "Slovenia"[Title/Abstract] OR "Spain"[Title/Abstract] OR "Sweden"[Title/Abstract] OR "Switzerland"[Title/Abstract] OR "Turkey"[Title/Abstract] OR "Ukraine"[Title/Abstract] OR "united kingdom"[Title/Abstract]<br><br>#1 AND #2 AND (#3 OR #4) AND #5

EmBase	#1. ('human immunodeficiency virus infection'/exp OR 'human immunodeficiency virus'/exp OR human immunodeficiency virus*:ti,ab,kw OR hiv:ti,ab,kw)<br><br>#2. (''pregnancy'/exp OR 'pregnant person'/exp OR pregnan*:ti,ab,kw OR gestation*:ti,ab,kw OR 'child bearing'/exp OR 'childbearing'/exp OR 'mother'/exp OR 'maternal welfare'/exp OR mother*:ti,ab,kw)<br><br>#3. ('cd4 lymphocyte count'/exp OR 'viral load'/exp OR 'medication compliance'/exp OR 'patient compliance'/exp OR 'antiretroviral therapy'/exp OR 'vertical infection'/exp OR 'virus drug resistance'/exp OR cd4:ti,ab,kw OR 'viral load':ti,ab,kw OR 'hiv rna':ti,ab,kw OR adherence:ti,ab,kw OR compliance:ti,ab,kw OR art:ti,ab,kw OR arv:ti,ab,kw OR 'vertical transmission':ti,ab,kw OR mtct:ti,ab,kw)<br><br>#4. ('social support'/exp OR 'mental health'/exp OR 'quality of life'/exp OR 'depression'/exp OR 'anxiety'/exp OR 'parenting'/exp OR 'family'/exp OR 'psychological stress'/exp OR 'social isolation'/exp OR 'social support':ti,ab,kw OR stigma:ti,ab,kw OR 'mental health':ti,ab,kw OR 'quality of life':ti,ab,kw OR depression:ti,ab,kw OR anxiety:ti,ab,kw OR coping:ti,ab,kw OR parenting:ti,ab,kw OR disclosure:ti,ab,kw OR family:ti,ab,kw OR 'psychological stress':ti,ab,kw OR 'social isolation':ti,ab,kw)<br><br>#5. ('europe'/exp OR europ*:ti,ab,kw OR 'albania'/exp OR albania:ti,ab,kw OR 'andorra'/exp OR andorra:ti,ab,kw OR 'armenia'/exp OR armenia:ti,ab,kw OR 'austria'/exp OR austria:ti,ab,kw OR 'azerbaijan'/exp OR azerbaijan:ti,ab,kw OR 'belarus'/exp OR belarus:ti,ab,kw OR 'belgium'/exp OR belgium:ti,ab,kw OR 'bosnia and herzegovina'/exp OR (bosnia:ti,ab,kw AND herzegovina:ti,ab,kw) OR 'bulgaria'/exp OR bulgaria:ti,ab,kw OR 'croatia'/exp OR croatia:ti,ab,kw OR 'cyprus'/exp OR cyprus:ti,ab,kw OR 'czech republic'/exp OR 'czech republic':ti,ab,kw OR 'denmark'/exp OR denmark:ti,ab,kw OR 'estonia'/exp OR estonia:ti,ab,kw OR 'finland'/exp OR finland:ti,ab,kw OR 'france'/exp OR france:ti,ab,kw OR 'georgia'/exp OR georgia:ti,ab,kw OR 'germany'/exp OR germany:ti,ab,kw OR 'greece'/exp OR greece:ti,ab,kw OR 'hungary'/exp OR hungary:ti,ab,kw OR 'iceland'/exp OR iceland:ti,ab,kw OR 'ireland'/exp OR ireland:ti,ab,kw OR 'italy'/exp OR italy:ti,ab,kw OR 'kazakhstan'/exp OR kazakhstan:ti,ab,kw OR 'latvia'/exp OR latvia:ti,ab,kw OR 'lithuania'/exp OR lithuania:ti,ab,kw OR 'luxembourg'/exp OR luxembourg:ti,ab,kw OR 'malta'/exp OR malta:ti,ab,kw OR 'moldova'/exp OR moldova:ti,ab,kw OR 'monaco'/exp OR monaco:ti,ab,kw OR 'montenegro'/exp OR montenegro:ti,ab,kw OR 'netherlands'/exp OR netherlands:ti,ab,kw OR 'north macedonia'/exp

	OR 'north macedonia':ti,ab,kw OR 'norway'/exp OR norway:ti,ab,kw OR 'poland'/exp OR poland:ti,ab,kw OR 'portugal'/exp OR portugal:ti,ab,kw OR 'romania'/exp OR romania:ti,ab,kw OR 'russia'/exp OR russia:ti,ab,kw OR 'san marino'/exp OR 'san marino':ti,ab,kw OR 'serbia'/exp OR serbia:ti,ab,kw OR 'slovakia'/exp OR slovakia:ti,ab,kw OR 'slovenia'/exp OR slovenia:ti,ab,kw OR 'spain'/exp OR spain:ti,ab,kw OR 'sweden'/exp OR sweden:ti,ab,kw OR 'switzerland'/exp OR switzerland:ti,ab,kw OR 'turkey'/exp OR turkey:ti,ab,kw OR 'ukraine'/exp OR ukraine:ti,ab,kw OR 'united kingdom'/exp OR 'united kingdom':ti,ab,kw)<br><br>#1 AND #2 AND (#3 OR #4) AND #5
Cochrane	#1 MeSH descriptor: [HIV] explode all trees<br>#2 MeSH descriptor: [HIV Infections] explode all trees<br>#3 ((human immunodeficiency virus OR hiv)):ti,ab,kw<br>#4 MeSH descriptor: [Pregnancy] explode all trees<br>#5 MeSH descriptor: [Pregnant People] explode all trees<br>#6 MeSH descriptor: [Mothers] explode all trees<br>#7 (pregnan* OR mother* OR childbearing OR "child bearing" OR child-bearing):ti,ab,kw<br>#8 MeSH descriptor: [CD4 Lymphocyte Count] explode all trees<br>#9 MeSH descriptor: [Viral Load] explode all trees<br>#10 MeSH descriptor: [Medication Adherence] explode all trees<br>#11 MeSH descriptor: [Patient Compliance] explode all trees<br>#12 MeSH descriptor: [Anti-Retroviral Agents] explode all trees<br>#13 MeSH descriptor: [Infectious Disease Transmission, Vertical] explode all trees<br>#14 MeSH descriptor: [Drug Resistance, Viral] explode all trees<br>#15 (cd4 OR "viral load" OR "hiv rna" OR adherence OR compliance OR art OR arv OR "vertical transmission" OR mtct):ti,ab,kw<br>#16 MeSH descriptor: [Social Support] explode all trees<br>#17 MeSH descriptor: [Mental Health] explode all trees<br>#18 MeSH descriptor: [Quality of Life] explode all trees<br>#19 MeSH descriptor: [Depression] explode all trees<br>#20 MeSH descriptor: [Anxiety] explode all trees<br>#21 MeSH descriptor: [Parenting] explode all trees<br>#22 MeSH descriptor: [Family] explode all trees<br>#23 MeSH descriptor: [Stress, Psychological] explode all trees<br>#24 MeSH descriptor: [Social Isolation] explode all trees<br>#25 MeSH descriptor: [Social Stigma] explode all trees<br>#26 ("social support" OR stigma OR "mental health" OR "quality of life" OR depression OR anxiety OR coping OR parenting OR disclosure OR family OR "psychological stress" OR "social isolation"):ti,ab,kw

	#27 MeSH descriptor: [Europe] explode all trees<br>#28 (Europe* OR Albania OR Andorra OR Armenia OR Austria OR Azerbaijan OR Belarus OR Belgium OR Bosnia and Herzegovina OR Bulgaria OR Croatia OR Cyprus OR Czech Republic OR Denmark OR Estonia OR Finland OR France OR Georgia OR Germany OR Greece OR Hungary OR Iceland OR Ireland OR Italy OR Kazakhstan OR Latvia OR Lithuania OR Luxembourg OR Malta OR Moldova OR Monaco OR Montenegro OR Netherlands OR North Macedonia OR Norway OR Poland OR Portugal OR Romania OR Russia OR San Marino OR Serbia OR Slovakia OR Slovenia OR Spain OR Sweden OR Switzerland OR Turkey OR Ukraine OR United Kingdom):ti,ab,kw<br>#29 (humans AND (female OR women) AND adult*):ti,ab,kw<br>#30 #1 OR #2 OR #3<br>#31 #3 OR #4 OR #5 OR #6 OR #7<br>#32 #8 OR #9 OR #10 OR #11 OR #12 OR #13 OR #14 OR #15<br>#33 #16 OR #17 OR #18 OR #19 OR #20 OR #21 OR #22 OR #23 OR #24 OR #25 OR #26<br>#34 #27 OR #28<br>#35 #30 AND #31 AND (#32 OR #33) AND #34 AND #29
Web of Science	#1. TS=("hiv" OR "hiv infections" OR "human immunodeficiency virus*")<br><br>#2. TS= (pregnan* OR gestation* OR "child bearing" OR childbearing OR mother*)<br><br>#3. TS=("CD4" OR "Viral Load" OR "HIV RNA" OR "adherence" OR "compliance" OR "ART" OR "ARV" OR "vertical transmission" OR "MTCT")<br><br>#4. TS=("Social Support" OR "Mental Health" OR "Quality of Life" OR "Depression" OR "Anxiety" OR "coping" OR "Parenting" OR "disclosure" OR "Family" OR "psychological stress" OR "Social Isolation" OR "stigma")<br><br>#5. TS = ("Europe" OR "Albania" OR "Andorra" OR "Armenia" OR "Austria" OR "Azerbaijan" OR "Belarus" OR "Belgium" OR "Bosnia and Herzegovina" OR "Bulgaria" OR "Croatia" OR "Cyprus" OR "Czech Republic" OR "Denmark" OR "Estonia" OR "Finland" OR "France" OR "Georgia" OR "Germany" OR "Greece" OR "Hungary" OR "Iceland" OR "Ireland" OR "Italy" OR "Kazakhstan" OR "Latvia" OR "Lithuania" OR "Luxembourg" OR "Malta" OR "Moldova" OR "Monaco" OR "Montenegro" OR "Netherlands" OR "North Macedonia" OR "Norway" OR "Poland" OR "Portugal" OR "Romania" OR "Russia" OR "San Marino" OR "Serbia" OR "Slovakia" OR "Slovenia" OR "Spain" OR "Sweden" OR "Switzerland" OR "Turkey" OR "Ukraine" OR "United Kingdom")

	#1 AND #2 AND (#3 OR #4) AND #5
ScienceDirect	Title, abstract or author-specified keywords<br>#1 (HIV OR "human immunodeficiency virus")<br>#2 (pregnant OR pregnancy OR mother OR motherhood OR maternal)<br>#3 (Europe OR European)<br><br>#1 AND #2 AND #3

Supplementary Table S2. Data charting and extraction of included studies

Title	Author	Year	Country	Study Aim	Methods and Outcome Measures	Population	Key findings
Missed opportunities among HIV-positive women to control viral replication during pregnancy and to have a vaginal delivery.	Aebi Popp	2013	Switzerland<br>Belgium<br>Denmark,<br>Germany<br>Italy<br>Netherlands<br>Poland<br>Spain<br>Sweden<br>United Kingdom	To explore the impact of guidelines recommending vaginal delivery for women with undetectable or very low viral load on delivery modes in Europe.	Pooled cohort analysis (Swiss and European studies, 2000–2010). Data included sociodemographics, ART timing, immunological and virological status, delivery mode, and gestational age.	2663 WLHIV	2,663 women with 3,013 deliveries were included from 10 countries.<br>28% were diagnosed with HIV during pregnancy.<br>cART was used in most pregnancies, starting during the first or second trimester in 78% and during the third trimester in 22%. In 25% of pregnancies, the woman conceived on cART.<br>In 86% of pregnancies, VL <400 copies/mL was achieved before delivery.<br>The proportion of vaginal deliveries increased from 17% (414/2,377) before the guideline change to 52% (313/600) after; elective caesarean section rates decreased from 65% to 27%.<br>he proportion of women with undetectable VL having a caesarean section was 55% after implementation of the new guidelines.<br>Late preterm deliveries decreased from 16% (377/2,354) before to 7% (42/599) after the guideline change.<br>There are still missed opportunities for women with HIV to fully suppress their viral load and to deliver vaginally in Europe.
Pregnancy and delivery outcomes of HIV infected women in Switzerland 2003-2008.	Aebi-Popp	2010	Switzerland	To describe maternal characteristics and their association with pregnancy outcomes in women living with HIV.	Prospective cohort study using anonymous questionnaires (2003–2008). Data included maternal clinical and laboratory measures, ART use, substance use, sociodemographic factors, and pregnancy outcomes (preterm birth, pre-eclampsia, gestational diabetes).	266 WLHIV	67 (25.2%) were first diagnosed with HIV during pregnancy.<br>28 of 266 women (10.5%) had a vaginal delivery, including 21 spontaneous deliveries, 6 vacuum extractions, and 1 forceps extraction, whereas 236 (88.7%) delivered by caesarean section.<br>Of the caesarean sections, 180 (67.7%) were elective, including 8 before 37 weeks' gestation. Secondary caesarean sections were performed in 56 cases (21.1%) to prevent MTCT or preterm labour.<br>All women undergoing vaginal deliveries had an HIV RNA viral load <50 copies/mL.<br>The majority of women (251; 94.4%) received ART during pregnancy. Of these, 172 (68.5%) were already on ART prior to pregnancy, while in 94 cases (35.5%) treatment was initiated during pregnancy regardless of CD4 cell count to prevent vertical transmission.<br>Diagnosis of HIV after conception was much more common among non-Caucasian women (83.6%) compared with Caucasian women (16.4%). 30% (n=80) of the women had pregnancy complications after 24 weeks' gestation.<br>Preterm delivery occurred in 72 (27%) patients. Other complications included pre-eclampsia (n=7; 2.6%) and gestational diabetes (n=7; 2.6%).<br>Older maternal age was the only risk factor significantly associated with adverse pregnancy outcomes.

Postnatal retention in HIV care: insight from the Swiss HIV Cohort Study over a 15-year observational period.	Aebi-Popp	2016	Switzerland	To quantify loss to follow-up in HIV care after delivery and identify associated risk factors and implications.	Cohort data (1996–2011). Outcomes included CD4 count, viral load, gestational age, delayed clinical attendance, and loss to follow-up postpartum.	580 WLHIV	A total of 695 pregnancies in 580 women were included in the study, of which 115 (17%) were subsequent pregnancies.<br>104 (15%) women reported a history of injecting drug use (IDU).<br>233 of 695 (34%) women had a delayed visit in the year after delivery, and 84 (12%) women were lost to follow-up. Being lost to follow-up was significantly associated with a history of IDU and not achieving an undetectable HIV viral load at delivery.<br>43 of 84 (55%) women returned to care after LTFU. Half of them (20 of 41) with available CD4 had a CD4 count <350 cells/µL and 15% (6 of 41) a CD4 count <200 cells/µL at return.
Neglect of attention to reproductive health in women with HIV infection: contraceptive use and unintended pregnancies in the Swiss HIV Cohort Study	Aebi-Popp	2018	Switzerland	To evaluate contraceptive use and unintended pregnancies among women living with HIV in Switzerland.	Cross-sectional questionnaire study on contraception and unintended pregnancy; supplemented with cohort database clinical data.	462 WLHIV	Of 462 women included, 164 (35.5%) reported not using any contraception.<br>Among these, 65 (39.6%) reported being sexually active, although 29 (44.6%) were not planning a pregnancy.<br>Of 298 women using contraception, the following methods were reported: condoms 219 (73.5%); oral hormonal contraception 32 (10.7%); and intrauterine devices 28 (9.4%).<br>Among all women using contraception, 32 (10.7%) reported using more than one contraceptive method and 48 (16%) had an unintended pregnancy while on contraception (18 condoms; 16 oral contraception; four other methods).<br>Of these, 68.1% terminated the pregnancy and almost half (43.7%) continued using the same contraceptive method after the event.<br>The most frequent ethnicity was Black African origin (44.4%).
Factors Associated with Non-disclosure of HIV Status in a Cohort of Childbearing HIV-Positive Women in Ukraine	Ahn	2016	Ukraine	To determine the prevalence and factors associated with non-disclosure of HIV status among recently delivered women.	Observational cohort with questionnaire and clinician data; outcomes included HIV status disclosure and associated factors (logistic regression).	2019 WLHIV	95% disclosed their HIV status to at least one person.<br>90% disclosed to their partner, 58% to at least one parent, 8% to other family members, and 2% to friends.<br>Non-disclosure was higher among cohabiting women, those not knowing their partner's HIV status, and less educated women.<br>Older age was associated with non-disclosure in unadjusted analyses.<br>Clinical factors (ART, CD4, WHO stage) and behavioural factors (IDU, smoking, alcohol) were not associated with disclosure.<br>One in ten had not disclosed to their partner, and one in 20 had disclosed to no one.
Most women living with HIV can deliver vaginally- National data from Finland 1993-2013.	Aho	2018	Finland	To assess national rates of vaginal delivery and indications for caesarean section among women living	Retrospective registry study; outcomes included ART status, CD4 count, viral load, delivery mode, and caesarean section indications.	212 WLHIV with 290 deliveries (including four pairs of twins).	Overall vaginal delivery rate 74.5%, elective caesarean section 12.8%, emergency caesarean section 12.8%.<br>80% of women had viral load <50 copies/mL before delivery.<br>Most caesarean sections were for obstetric reasons (63.5%), HIV avoidance (28.4%), or maternal request (0.7%).<br>Vaginal delivery rate increased in subsequent deliveries (72.7% to 82.2%).<br>Hospitals with fewer HIV deliveries had higher elective caesarean section rates (22.7% vs 10.6%).<br>No perinatal HIV transmissions occurred.

				with HIV in Finland.			
Role of HIV in the desire of procreation and motherhood in women living with HIV in Spain: a qualitative approach	Alvarez-del Arco	2018	Spain	To analyse factors influencing reproductive desire and the impact of HIV.	Qualitative interviews exploring experiences, stigma, and perceptions of motherhood.	20 WLHIV	HIV diagnosis is usually associated with fear of immediate death and the idea of social isolation. At this moment, women may temporarily reject the idea of future motherhood or having a sexual life.<br>HIV status is often disclosed only to a close social circle, and partner support is essential in the process of assimilating the diagnosis.<br>Health professionals provide information on assisted reproductive technology and on how to minimise the risk of partner HIV transmission.<br>Most barriers to procreation acknowledged by women are not directly related to HIV; however, women fear vertical transmission and face other challenges derived from the infection.<br>In this context, pregnancy allows women to feel "normal" despite HIV.<br>Motherhood is considered a compensatory element that helps them cope with the diagnosis.
Induced first abortion rates before and after HIV diagnosis: results of an Italian self-administered questionnaire survey carried out in 585 women living with HIV.	Ammas sari	2013	Italy	To investigate the impact of HIV diagnosis on reproductive planning and abortion predictors.	Questionnaire-based study capturing sociodemographic, behavioural, reproductive, and clinical data; statistical analysis conducted.	585 WLHIV	The crude incidence rate of abortion was 18.8 per 1,000 person-years of follow-up (PYFU).<br>Compared with women who terminated their pregnancy before HIV diagnosis, women who terminated their pregnancy after HIV diagnosis but before 1990 showed a 2.56-fold higher risk.<br>During 1990–1999 and 2000–2010, HIV diagnosis was not significantly associated with outcome (adjusted rate ratio 0.69).<br>Age and injecting drug use were predictors of abortion overall.<br>After HIV diagnosis, being on combination antiretroviral therapy, monthly income <€800, younger age, and fear of vertical transmission were independently associated with abortion.<br>There was a higher incidence of abortion compared with data from the general Italian population.<br>Awareness of HIV diagnosis was predictive of abortion only in the 1980s.<br>Women with HIV infection remain concerned about vertical HIV transmission.
Term labor management and outcomes in treated HIV-infected women without contraindications to vaginal delivery and matched controls	Azria	2010	France	To assess labour management and outcomes in treated HIV-positive pregnant women.	Retrospective case–control study comparing women with and without HIV; outcomes included labour, delivery, and neonatal outcomes.	146 WLHIV and 146 women without HIV	The mode of delivery was similar in the two groups, but the episiotomy rate was significantly lower among HIV-infected women (29.6% vs 45.6%, $p = 0.01$), with no difference in mean birth weight, simple or complex perineal laceration rates, or neonatal outcomes.<br>Postpartum morbidity was also similar for controls and HIV-infected women with a CD4+ cell count of 200 cells/mL or higher.<br>However, in the study group, postpartum morbidity was higher among those with a CD4+ cell count below this threshold (3.2% vs 22.2%, $p = 0.007$).<br>No case of mother-to-child transmission of HIV occurred.<br>HIV-infected women with no contraindication to vaginal delivery appear to have similar labour outcomes to uninfected women

Pregnancy outcomes in HIV-positive women in Ukraine, 2000-12 (European Collaborative Study in EuroCoord): an observational cohort study	Bagkeris	2015	Ukraine	To assess birth outcomes and risk factors in women living with HIV in Ukraine.	Prospective cohort (European Collaborative Study); outcomes included preterm birth and small-for-gestational-age infants (Poisson regression).	8,884 WLHIV and liveborn infant pairs	Median maternal age 26.5 years.<br>832 (11%) women had WHO stage 3 or 4 HIV.<br>1,474 (17%) had a history of injecting drug use.<br>7,348 (83%) received antenatal ART (4,396 zidovudine monotherapy, 2,949 combination ART).<br>Preterm delivery occurred in 780 (9%) of 8,860 births; 77 (9%) of 889 babies were small for gestational age.<br>Factors associated with preterm delivery: injecting drug use, no ART, antenatal combination ART, WHO stage 4 HIV, and most socially deprived group.<br>Small for gestational age associated with injecting drug use, social deprivation, no ART, and antenatal combination ART.<br>Some risk factors were directly associated with HIV and treatment; others were shared with the general antenatal population.<br>Monitoring of pregnancy outcomes is important as antenatal combination ART use increases.
Adherence to antiretroviral therapy during pregnancy and the first year postpartum among HIV-positive women in Ukraine	Bailey	2014	Ukraine	To assess adherence to antiretroviral therapy during pregnancy and postpartum and identify at-risk groups.	Cross-sectional surveys measuring ART adherence antenatally and postpartum using validated tools and self-report.	287 WLHIV	185 antenatal and 102 postnatal.<br>During pregnancy, 14% had a CASE score ≤11 and 35% reported ≥1 missed dose; postnatally 8% had a CASE score ≤11 and 31% reported ≥1 missed dose.<br>Poorer adherence was associated with younger age, unplanned pregnancy, living with extended family, low self-efficacy, smoking, non-disclosure, and history of illicit drug use.<br>Most women worried about ART harming the baby despite believing in its effectiveness.<br>Results highlight unmet needs for counselling and support.<br>Some groups are at risk of poor ART adherence, including women with markers of social vulnerability and those with low ART-related self-efficacy.
Factors associated with abandonment of infants born to HIV-positive women: results from a Ukrainian birth cohort	Bailey	2010	Ukraine	To quantify rates of infant abandonment and associated factors in Ukraine.	Prospective cohort with follow-up to 24 months; outcome: infant abandonment.	4759 WLHIV–infant pairs	2.1% of infants abandoned by age 2; additional 1% in partial non-parental care.<br>Abandonment declined from 3.8% (2000–2002) to 1.6% (2006–2009).<br>Protective factors: antenatal ART, higher maternal education, being married/cohabiting, elective caesarean delivery.<br>Risk factors: active maternal IDU, higher parity, preterm birth <34 weeks, no cohabiting partner or husband.<br>Abandoned infants more likely HIV-infected and had higher mortality.<br>Women delivering by elective caesarean section were less likely to abandon, as were those leaving full-time education later.<br>A decline in infant abandonment over the last 10 years was seen in this cohort, concurrent with PMTCT scale-up, suggesting the importance of PMTCT programmes in reducing infant abandonment rates among HIV-infected women.

							However, the challenge of ensuring coverage of hard-to-reach populations such as IDUs and other marginalised groups with PMTCT services remains.
Impact of expanded access to combination antiretroviral therapy in pregnancy: results from a cohort study in Ukraine	Bailey	2013	Ukraine	To investigate the scale-up of antenatal antiretroviral therapy in Ukraine.	Cohort analysis examining ART regimens and mother-to-child transmission rates.	3535 pregnant WLHIV	cART coverage increased significantly, from 22% of deliveries in 2008 to 61% of those in 2010.<br>Initiation of antenatal AZTm – rather than cART – was associated with cohabiting (versus being married), at least two previous live births (versus none) and a diagnosis of HIV infection during the first or second trimester (versus before pregnancy).<br>The overall MTCT rate was 4.1%; 42% (49/116) of the transmissions were from the 8% (n=238) of women without antenatal ART.<br>Compared with AZTm, cART was associated with a 70% greater reduction in the risk of MTCT.
Prevalence of depressive symptoms in pregnant and postnatal HIV-positive women in Ukraine: a cross-sectional survey	Bailey	2016	Ukraine	To examine the burden and correlates of perinatal depression among women living with HIV.	Cross-sectional surveys assessing perinatal depression (PHQ-2) and associated psychosocial factors.	408 WLHIV	180 pregnant women (antenatal survey), 228 postpartum women (postnatal survey).<br>27% of antenatal and 25% of postnatal participants screened positive for depressive symptoms.<br>Risk factors antenatally: living alone, low ART self-efficacy, bothered by ART side effects.<br>Postnatally: single mothers, low confidence in neonatal prophylaxis, worried about harm from prophylaxis, low help-seeking self-efficacy.<br>Unmet need for mental health support: 31% of antenatal vs 82% of postnatal women wanting help were already accessing support services.<br>A quarter of women screened positive for depression.<br>Results highlight the need for proactive strategies to identify depressive symptoms, and an unmet need for provision of mental health support in the perinatal period for HIV-positive women in Ukraine.
Pregnancy outcomes in women with advanced HIV infection in Italy.	Baroncelli	2011	Italy	To compare maternal outcomes by stage of HIV disease and assess treatment response.	Multicentre observational study; outcomes included maternal and neonatal outcomes by HIV disease stage.	566 WLHIV	515 in CDC-AB group and 51 in CDC-C group.<br>Baseline characteristics were similar; median age 34 years.<br>Coinfections (HBV, HCV, CMV, HPV, STDs) were similar.<br>Unintended pregnancy was more common in CDC-C group.<br>No differences were found in main maternal and neonatal outcomes between CDC-C and CDC-AB groups.<br>Most women achieved viral suppression at the end of pregnancy.<br>One year after delivery, HIV replication was present in 30.0% of CDC-C women and 11.5% of CDC-AB women.<br>CD4 counts increased significantly from preconception to one year postpartum in both groups.<br>Only one new AIDS-defining event occurred in a CDC-C woman (non-Hodgkin's lymphoma), who died 4 months postpartum.<br>Preterm delivery and median gestational age were similar.<br>Neonatal outcomes, including birthweight, APGAR scores, HIV infection (three cases in CDC-AB), birth defects, and neonatal deaths were comparable between groups.

							Overall, pregnancy in CDC-C women was not associated with increased adverse maternal or neonatal outcomes.
Full Viral Suppression, Low-Level Viremia, and Quantifiable Plasma HIV-RNA at the End of Pregnancy in HIV-Infected Women on Antiretroviral Treatment	Baroncelli	2015	Italy	To determine rates of viral suppression in late pregnancy and associated determinants.	Observational study using plasma samples; outcome: viral suppression at late pregnancy.	107 pregnant WLHIV	According to plasma HIV-RNA levels, 3 groups were defined: full suppression (target not detected), low-level viraemia (target detected but <37 copies/ml), and quantifiable HIV-RNA (≥37 copies/ml).<br>Among 107 women evaluated at a median gestational age of 35 weeks, 90 (84.1%) had HIV-RNA <37 copies/ml.<br>Most of them (59/90, 65.6%) had full suppression, with the remaining (31/90, 34.4%) showing low-level viraemia.<br>Among the 17 women with quantifiable viral load, median HIV-RNA was 109 copies/ml, with only one case showing resistance.<br>Women with higher baseline HIV-RNA levels and with hepatitis C virus (HCV) coinfection were significantly more likely to have quantifiable HIV-RNA in late pregnancy.<br>Full viral suppression was significantly more likely with non-nucleoside reverse transcriptase inhibitor (NNRTI)-based regimens and significantly less likely with higher HIV-RNA in early pregnancy.<br>No cases of HIV transmission occurred.<br>HIV-infected pregnant women showed a high rate of viral suppression and a low resistance rate before delivery.<br>In most cases no target HIV-RNA was detected in plasma, suggesting a low risk of subsequent virological rebound and development of resistance.<br>Women with high levels of HIV-RNA in early pregnancy and those who have concomitant HCV infection should be considered at higher risk of having quantifiable HIV-RNA at the end of pregnancy.
Natural Conception is Safe for HIV-Serodiscordant Couples with Persistent Suppressive Antiretroviral Therapy for the Infected Partner.	Baza	2019	Spain	To evaluate reproductive counselling protocols for serodiscordant couples.	Descriptive cohort of serodiscordant couples; outcomes included conception success and pregnancy outcomes.	214 HIV serodiscordant couples<br>HIV positive man and HIV negative woman= 173<br>HIV negative man and HIV positive woman= 41	After almost 10,000 sexual relations, 188 pregnancies were achieved.<br>62% of couples became pregnant once or several times with no HIV transmission to either the partner or the offspring.<br>Younger age of woman, no fertility disorders in both members of the couple, and no treatment with efavirenz in men were factors related with reproductive success.<br>Natural conception under controlled conditions can be offered to serodiscordant couples (SDCs) as a safe method of conception, with effectiveness related to factors similar to those in the general population.

Trends in management and outcomes of pregnant women living with HIV between 2008-2013 and 2014-2019: A retrospective cohort study	Brandon	2022	United Kingdom	To assess maternal, virological, and perinatal outcomes over time.	Retrospective cohort study; outcomes included HIV management, delivery outcomes, and neonatal outcomes.	94 WLHIV	116 pregnancies.<br>Rate of preconception HIV diagnosis increased from 73% to 90%, and the proportion of WLHIV on cART at conception increased from 54% to 84%.<br>The median gestation at which cART was initiated antenatally decreased from 22+1 to 17+1 weeks.<br>In 2014–2019, 41% of WLHIV received non-nucleoside reverse transcriptase inhibitor-based cART, 37% protease inhibitor-based cART, and 22% of cART regimens contained an integrase inhibitor.<br>66% of WLHIV delivered by caesarean section, with a significant decrease over time in the rate of both planned and actual elective caesarean deliveries.<br>Perinatal outcomes included one case of perinatal HIV transmission (0.86%), 11% preterm birth, 15% small-for-gestational-age, and 2% stillbirth.<br>There was an association between a viral load >50 copies/mL at delivery and preterm delivery ($p = 0.0004$).<br>Virological, obstetric, and perinatal outcomes of WLHIV improved during the study period.
Pregnancy incidence and outcomes in women with perinatal HIV infection	Byrne	2017	United Kingdom	To estimate pregnancy incidence in women with perinatally acquired HIV and compare outcomes.	Population-based surveillance study; outcomes included pregnancy incidence, CD4 count, and delivery outcomes.	748 WLHIV	630 women with PHIV and 118 with BHIV.<br>Of the 630 women with PHIV, 7% (45) had at least one pregnancy, with 70 pregnancies reported.<br>The BHIV comparison group comprised 118 women with 184 pregnancies.<br>Women with PHIV were more likely to be on cART at conception and have a lower baseline CD4+ cell count.<br>PHIV and a low baseline CD4+ cell count were risk factors for detectable VL near delivery; older age at conception and being on cART at conception reduced this risk.<br>Women with PHIV in the United Kingdom have a low pregnancy incidence, but those who become pregnant are at risk of detectable VL near delivery, reflecting their often complex clinical history, adherence, and drug resistance issues.
Assessment of Pregnancy Status in Patients with Acquired Immunodeficiency Syndrome and their Partners	Cabalak	2024	Turkey	To investigate fertility status and related challenges among people living with HIV.	Cross-sectional observational study; outcomes included demographic and clinical characteristics.	261 adults with HIV<br>21 pairs where both partners were HIV positive<br>10 pairs where the woman was HIV positive	8 couples with both partners HIV positive had 9 children, and 6 serodiscordant couples with HIV-positive women had 10 children. The 5 serodiscordant couples with HIV-positive men had 6 children.During the follow-up period, no seroconversion was detected in the HIV-negative partners of serodiscordant couples.<br>Some pregnant women were first diagnosed during pregnancy<br>Most deliveries were by cesarean section; one vaginal delivery occurred at an outside center.<br>5 women had negative viral loads at delivery; 2 had positive viral loads.<br>No vertical transmission and no seroconversion in partners of serodiscordant couples.

						and the man was seronegative<br>5 pairs where the man was HIV positive and the woman was seronegative	
Clinical and Biological Risk Factors Associated with Increased Mother-to-Child Transmission of HIV in Two South-East HIV-AIDS Regional Centers in Romania	Cambrea	2022	Romania	To assess risk factors for mother-to-child transmission of HIV.	Retrospective cohort study; outcomes included mother-to-child transmission and predictors.	408 pregnant WLHIV	Comparing different variables of HIV-positive pregnant women from the two HIV-AIDS CRs: significant differences between the mean value of hemoglobin, CD4 level, environmental area, marital and amniotic membranes status, and HIV patient stage in the last trimester of pregnancy ($p < 0.05$), but without any differences in mother's mean age, education level, type of delivery, breastfeeding, the duration of cART administration, HIV viral load, and survival rate.<br>The most important clinical and biological risk factors associated with increased MTCT of HIV are represented by anemia, CD4 level, and HIV patient stage.

Pregnancy outcomes of women with HIV in a district general hospital in the UK.	Carey	2018	United Kingdom	To describe obstetric and virological outcomes in HIV-positive pregnant women.	Retrospective note review; outcomes included maternal HIV characteristics, pregnancy outcomes, and infant HIV status.	116 WLHIV	137 pregnancies in 116 women, with 136 live births.<br>Median maternal age 33 years.<br>Majority (82%, 112/136) Black African origin.<br>80% knew HIV status prior to pregnancy.<br>30% had history of AIDS-defining illness.<br>Social concerns included alcohol use (27% occasional, 43% moderate, 4% heavy), child protection concerns (16%), and history of domestic violence (27%).<br>60% of pregnancies unplanned.<br>Most pregnancies (84%) were uncomplicated.<br>71% achieved planned mode of delivery. Most common delivery mode: spontaneous vaginal delivery (42%), emergency Caesarean section (32%).<br>Gestational age ≥37 weeks in 84% of cases.<br>Maternal HIV VL at or closest to delivery: undetectable (<40 copies/mL) in 73%, <400 copies/mL in 90%, >1000 copies/mL in 6%.<br>About half of women were on HAART at conception; most not on HAART started treatment during pregnancy (90% PI-based).<br>Adherence self-reported as excellent in 18%, good in 73%, poor in 9%.<br>No infants were HIV-infected (MTCT 0%).
Sexuality and childbearing as it is experienced by women living with HIV in Sweden: a lifeworld phenomenological study	Carlsson-Lalloo	2018	Sweden	To explore experiences of sexuality and childbearing among women living with HIV.	Phenomenological qualitative study using in-depth interviews and thematic analysis.	18 WLHIV	The period immediately following diagnosis is described by many women as very difficult. Over time, many women begin a process of understanding and acceptance of the diagnosis. Re-creating sexuality and childbearing involves learning to cope with the virus so that these aspects of life are not controlled by perceptions of being contagious<br>One aspect of perceptions about HIV and its contagiousness is that the risk of transmission imposes both juridical and moral responsibility in relation to sexuality and childbearing. Regulations relating to HIV are described by the women as existing for a reason but are experienced as placing responsibility for transmission on the person living with HIV, generating feelings of guilt and shame.<br>The perceived contagiousness of HIV limits sexuality and childbearing and therefore influences sexual habits and considerations in relation to pregnancy and childbirth. These limitations are expressed through feelings of not having the possibility to choose freely in relation to sexuality and childbearing.<br>A higher level of knowledge about HIV transmission provides the ability to make confident choices and decisions about sexual habits, pregnancy, and childbirth. With deeper knowledge and understanding, women are able to make conscious decisions, which is important for feeling safe about what is and is not considered a risk of transmission.

Investigating the Pregnancy and Post-Partum Health Experiences of Women Living with HIV.	Cooper	2024	United Kingdom	To explore postpartum healthcare experiences and engagement in care.	Qualitative interview study (UK); thematic analysis of postpartum experiences.	11 WLHIV	Nine were of sub-Saharan African origin, one European, and one from the UK. All were taking antiretrovirals when they conceived and engaged with HIV services during their pregnancy. They had a total of 14 pregnancies, as three women gave birth twice in the seven-year period. Two had a detectable viral load, and one had no viral load data in the first 12 months postpartum. All pregnancies resulted in single live births with no vertical transmission of HIV. All women had been diagnosed with HIV prior to their pregnancy. None chose to breastfeed.<br>The three main themes were ‘infant feeding decisions’, ‘managing the risk of mother-to-child transmission’, and ‘managing the knowledge of their HIV status’.<br>All women struggled with the knowledge and decision not to breastfeed, often despite a strong personal desire to do so. They were acutely aware of the stigma surrounding not breastfeeding in their community<br>The transmission risk remained poorly understood by many women, creating further anxieties and remaining the greatest concern for all of the women.<br>An underlying theme running through all interviews was the stigma relating to HIV and fear of disclosure of their HIV status, revealing the depth of the social struggles and emotional impact of the stigma surrounding HIV. The majority of the women involved with this study had disclosed their HIV status to only a few people, so suddenly having new members of the medical team aware of their HIV diagnosis became very daunting<br>The anxieties of people learning of their HIV status was heightened during the postpartum period; women described several situations they believed posed a disclosure risk
Successful implementation of new Swiss recommendations on breastfeeding of infants born to women living with HIV	Crisinel	2023	Switzerland	To describe breastfeeding practices following guideline changes.	Cohort study of breastfeeding under clinical criteria; questionnaire on motivations.	20 WLHIV	The three main motivational factors were bonding, neonatal health benefits and maternal health benefits<br>Median breastfeeding duration was 6.3 months<br>None of the breastfed neonates received HIV post-exposure prophylaxis.<br>There was no HIV transmission: 24 infants tested negative for HIV at least three months after weaning, and one mother was still breastfeeding when the data were analysed.<br>As a result of a shared decision-making process, a high proportion of mothers expressed a desire to breastfeed.<br>Ongoing surveillance of breastfeeding mother–infant pairs in high-resource settings is needed to inform updates of guidelines and recommendations.

Natural conception in HIV-serodiscordant couples with the infected partner in suppressive antiretroviral therapy: A prospective cohort study.	Del Romero	2016	Spain	To evaluate reproductive counselling for natural conception in serodiscordant couples.	Prospective cohort of serodiscordant couples; outcomes included conception rates and HIV transmission.	161 HIV serodiscordant couples HIV-infected man and HIV seronegative woman (n=133 couples) HIV-infected woman and HIV seronegative man (n=28 couples)	Of the 161 couples, 107 (66%) achieved at least 1 pregnancy, 29 (18%) a second, and 8 (5%) a third.<br>Pregnancy rates per 100 acts of vaginal intercourse were 1.9 overall: 1.8 among HIV-seronegative women and 2.2 among HIV-infected women.<br>Mean time to achieve pregnancy was 6.1 months.<br>A total of 144 pregnancies occurred: 105 carried to term, 30 spontaneous abortions, 4 voluntary interruptions, 5 unknown outcomes.<br>A total of 107 infants were born, including 2 sets of twins.<br>Among 53 couples with clinical-analytic disorders affecting fertility, 32 pregnancies were achieved.<br>Some couples achieved pregnancy naturally after previous assisted reproduction failures.<br>No case of sexual or vertical HIV transmission occurred.<br>In the absence of fertility problems and under controlled conditions, natural conception might be a safe and effective eproductive method for those HIV serodiscordant couples who choose this reproductive option.
Residential and healthcare mobility during pregnancy among women living with HIV in the UK, 2009-2019.	Dema	2024	United Kingdom	To assess healthcare mobility during pregnancy and associated outcomes.	Observational study using national surveillance data; outcomes included residential mobility and viral load at delivery.	10,305 pregnancies	Among the included 10,305 pregnancies, 84.4% (8,695/10,305) of mothers were born outside the UK (of whom most [7,331/8,584] were born in sub-Saharan Africa).<br>Median age at delivery was 33 years, and in two-thirds of pregnancies the woman had experienced at least one previous pregnancy (ranging from 1 to 7).<br>Most pregnancies were conceived on ART, although in 3,744 (37.4%) women ART started antenatally, including 1,674 with an antenatal HIV diagnosis.<br>There were 89 stillbirths and 10,216 live births.<br>Among 10,305 pregnancies, 19.6% experienced residential mobility, 8.1% changed NHS Trust, and 4.5% changed SHA during pregnancy.<br>Mobility was more likely to be experienced by younger women, migrants, and those with a new antenatal diagnosis; residential but not healthcare mobility declined over time.<br>In a fully adjusted model, mobility was not associated with having a detectable viral load at delivery.<br>Higher proportions of infants were lost to follow-up after mobile pregnancies than after non-mobile pregnancies.

Missed opportunities to prevent mother-to-child transmission of HIV in Italy	Di Biagio	2019	Italy	To identify missed opportunities for preventing mother-to-child transmission.	Registry-based study of paediatric HIV cases; outcomes included missed prevention opportunities.	79 children vertically infected with HIV	Mothers came from different regions of the world: 32 were from Italy and 41 were born abroad, mainly in sub-Saharan Africa (n = 21) and Eastern Europe (n = 13). Risk factors for acquiring HIV were sexual intercourse (n = 32), injecting drug use (n = 10), blood transfusion (n = 1), and unknown (n = 36).<br>9 women were aware of their HIV infection before pregnancy, while 25 were diagnosed during pregnancy or in the peripartum period. In 12 women whose initial HIV test was negative, acute infection developed during pregnancy; only 5 were retested and diagnosed before delivery. In 32 women, HIV status remained unknown until the postpartum period. Only 15 of 19 women with known HIV infection received ART during pregnancy, and 3 stopped treatment before delivery. Among the 34 women diagnosed before delivery, only 17 received intravenous ZDV during labour.<br>Among the 79 children, 38 were born by vaginal delivery, 28 by elective caesarean section, 6 by caesarean section after prolonged labour, and 3 by spontaneous delivery. After birth, 31 infants received ZDV, and 39 were breastfed, including 9 despite known maternal HIV infection.<br>Missed opportunities occurred in 66 of the 79 women: 37 were not tested during pregnancy, and 3 with known HIV missed ART. Among the 34 women diagnosed before delivery, 11 missed the opportunity for caesarean section, 18 missed intrapartum ZDV, and 9 missed formula feeding. Additionally, 8 of 34 newborns did not receive ZDV in the first 4 weeks of life; only 3 received combination ART (ZDV + lamivudine + nevirapine).
Interdisciplinary correlations regarding the clinical and paraclinical evaluations in HIV-positive pregnant women.	Dorobat	2014	Romania	To assess interdisciplinary care in managing pregnancy in women living with HIV.	Clinical observational study; outcomes included delivery characteristics and neonatal health indicators.	36 WLHIV	Birth weight was less than 2,700 grams in all newborns of HIV-positive pregnant women or those with advanced disease (AIDS)<br>The primordial desideratum is to decrease the rate of mother-fetus vertical transmission, thus the caesarian section has been established as the birth method in all HIV-positive pregnant women after 38 weeks of amenorrhea, on intact membranes, outside labor, resulting in halving the percentage of HIV-positive children.<br>A very important role belongs to the interdisciplinary collaboration between the obstetrician and the infectious diseases specialist during the pregnancy, but also during the postpartum period. The role of the obstetrician is present in all the moments of pregnancy evolution. The HIV-positive pregnant woman is included in the group of high risk pregnancies.

Factors associated with HIV RNA levels in pregnant women on non-suppressive highly active antiretroviral therapy at conception	European Collaborative Study	2010	Western Europe - Not specified	To analyse viral load patterns in pregnancy.	Prospective cohort; outcomes included HIV RNA trends and transmission.	127 WLHIV on HAART	HIV RNA levels remained roughly constant until 10 weeks gestation, then declined by ~0.06 log10 copies/ml per week until delivery.<br>Women with baseline CD4 <500 cells/mm³ had higher HIV RNA level<br>NNRTI-based HAART at conception was associated with consistently lower VL than PI-based HAART.<br>62% of women had detectable VL within 4 weeks of delivery (median 2.40 log10 copies/ml).<br>MTCT rate was low: 1.27% overall.<br>Treatment interruptions and regimen switches were common early in pregnancy; most women achieved partial virological control during pregnancy.<br>Variety of practices regarding the management of women conceiving on HAART with detectable VL in this Western European setting. Clinical concerns during pregnancy include attempts to improve virological control to avoid MTCT and improve maternal health, whilst minimising potential adverse effects on the foetus and mother, including the risk of exposure to potentially teratogenic drugs.
Mode of delivery in HIV-infected pregnant women and prevention of mother-to-child transmission: changing practices in Western Europe	European Collaborative Study	2010	Belgium<br>United Kingdom<br>Netherlands<br>Italy<br>Spain<br>Germany<br>Denmark<br>Sweden	To examine delivery mode trends and transmission risk.	Cohort study; outcomes included maternal and infant HIV characteristics.	5,238 WLHIV mother–child pairs	The elective CS rate increased from 16% in 1985–1993 to 67% in 1999–2001, declining to 51% by 2005–2007.<br>Vaginal deliveries accounted for 10% of births in 2002–2004, increasing to 34% by 2005–2007<br>During the HAART era, women in Belgium, the United Kingdom, and the Netherlands were less likely to deliver by elective CS than those in Italy and Spain. The MTCT rate in 2005–2007 was 1%.<br>Among MCPs with maternal HIV RNA <400 copies/mL (n = 960), elective CS was associated with an 80% decreased MTCT risk , adjusting for HAART and prematurity.<br>Two infants born to 559 women with viral loads <50 copies/mL were infected, one delivered by elective CS (MTCT rate 0.4%; 95% CI 0.04–1.29).<br>Elective CS appears to prevent MTCT even at low maternal viral loads, although the study was underpowered to determine whether this applies for viral loads <50 copies/mL.<br>Diverging patterns of mode of delivery in Europe reflect uncertainties regarding the risk–benefit balance of elective CS for women on successful HAART.

Migrant women living with HIV in Europe: are they facing inequalities in the prevention of mother-to-child-transmission of HIV?	Favarato	2018	Belgium<br>Denmark<br>Germany<br>Italy<br>Netherlands<br>Poland<br>Sweden<br>United Kingdom<br>Ireland<br>Spain	To examine barriers to HIV testing and care among migrant pregnant women.	Pooled cohort analysis examining PMTCT interventions among migrant vs non-migrant women.	9421 WLHIV	Data included 9,421 (79.9%) migrant women, mainly from sub-Saharan Africa (SSA);<br>4,134 migrant women were diagnosed in the current pregnancy, often (48.6%) presenting with CD4 count <350 cells/ml.<br>In Europe a large proportion of pregnant women with HIV are migrants—mainly from sub-Saharan Africa,<br>Migrant women are less likely than native-born women to know their HIV status at conception, more likely to be diagnosed in late pregnancy and at antenatal diagnosis, and more likely to have low CD4 counts.<br>Among those on ART there is no overall difference between migrant and native women with respect to achieving an undetectable viral load by delivery
Stillbirth in Women Living With HIV Delivering in the United Kingdom and Ireland: 2007-2015	Favarato	2019	United Kingdom<br>Ireland	To describe stillbirth rates and associated risk factors.	Population-based surveillance study; outcomes included stillbirth rates and associated factors.	8090 WLHIV	There were 10,434 singleton deliveries, 89% (9,275) of which occurred in England and Wales.<br>Most pregnancies were in women of African origin (7,781/10,317, 75.4%), with East Africa being the most common region of origin (4,319/10,317, 41.9%).<br>Overall, 48.6% (5,023/10,328) of pregnancies were conceived on ART, and in 34.4% (3,381/9,830) of pregnancies, baseline CD4 count was ≤350 cells/mm³.<br>Most pregnancies were in parous women (7,221/9,926, 72.8%), and the median age at delivery was 32.7 years (interquartile range 28.6–36.4).<br>Eighty-nine singletons were stillborn, equivalent to a rate of 8.5 per 1,000 births.<br>Over the study period, the stillbirth rate tended to decline, but the temporal trend was not statistically significant.<br>Most stillbirths were delivered preterm compared with 1,105/10,345 (10.7%) among live births, and more than half occurred at <34 weeks compared with 410/10,345 (4.0%) of live births.<br>Among stillborn infants, 58.2% (39/67) were male and 50.0% (31/62) were small for gestational age.<br>Congenital anomalies were reported in 16.1% (10/62) of stillborn infants compared with 2.9% (286/10,026) of live-born infants<br>Risk factors for stillbirth included pre-eclampsia, diabetes, Asian maternal origin (versus United Kingdom/Ireland), CD4 count <350 cells/mm³, older maternal age, and primiparity.<br>Conceiving on ART did not increase the risk.<br>The standardised stillbirth ratio was 129 (95% confidence interval: 101 to 165) in WLHIV compared with the general population.

Rate, Predictors, and Consequences of Late Antenatal Booking in a National Cohort Study of Pregnant Women With HIV in Italy	Floridia	2014	Italy	To evaluate late antenatal booking and its consequences.	Observational study; outcomes included antenatal care timing and pregnancy outcomes.	1,643 pregnancies among WLHIV	1,674 live births<br>Rate of late booking among 1,643 pregnancies was 32.9%.<br>Overall, one-third of the women were of African origin, and the most common route of transmission was represented by sexual contacts (74.9%).<br>Mothers were usually ART-experienced and diagnosed with HIV before pregnancy, with a good immunological status, and a very infrequent history of past clinical AIDS-defining events.<br>Women with a late booking were usually younger, were more commonly of African provenance, and were much more frequently diagnosed with HIV during current pregnancy. As a consequence, ART exposure both before and during pregnancy was significantly lower in the women of this group; they also less frequently had a personal indication to ARTand a previous history of clinical events defining HIV disease stage C.<br>Undetectable HIV RNA at third trimester and preterm delivery were significantly more prevalent with earlier booking, whereas complications of delivery were more common with late booking<br>Multivariable analyses confirmed an independent role of late booking in predicting detectable HIV RNA at third trimester and delivery complications
Consequences of Presentation With Advanced HIV Disease in Pregnancy: Data From a National Study in Italy	Floridia	2015	Italy	To assess determinants and outcomes of advanced HIV disease in pregnancy.	National observational study; outcomes included CD4 count, maternal and neonatal outcomes.	469 WLHIV	Pregnant women diagnosed with HIV during pregnancy<br>15.8% (74/469) presented with advanced HIV disease (CD4 <200).<br>African origin was the only significant predictor of advanced disease<br>5.6% of women with CD4 <200 developed severe AIDS-defining conditions; none occurred in women with higher CD4 (P<0.001).<br>Early preterm birth (<32 weeks) more frequent with low CD4 (6.2% vs. 1.4%, P=0.015).<br>One case of vertical HIV transmission occurred in the low-CD4 group.<br>Emphasis on earlier HIV testing, particularly for women of African origin, to prevent morbidity.<br>Earlier access to HIV testing, particularly among immigrants of African origin, can prevent severe HIV-related morbidity.

Pregnant with HIV before age 25: Data from a large national study in Italy, 2001-2016	Floridia	2017	Italy	To determine prevalence of undiagnosed HIV and adverse outcomes in young pregnant women.	Longitudinal cohort analysis; outcomes included temporal trends in HIV care and pregnancy outcomes.	2979 WLHIV	2979 pregnancies.<br>Median age 33 years.<br>9.0% (269/2979) of pregnancies were in women <25 years.<br>Younger women more frequently of foreign origin (68.2% vs. 45.6%).<br>Younger women had lower rate of planned pregnancy, were more frequently diagnosed with HIV in pregnancy, and if already diagnosed before pregnancy, were less frequently on ARV at conception.<br>During pregnancy, treatment coverage was almost universal in both age groups, with no differences in HIV viral suppression at third trimester or adverse pregnancy outcomes.<br>Young women represent a growing proportion of pregnant women with HIV and are more likely to have unplanned pregnancy, undiagnosed HIV, and lower treatment coverage at conception.
Pregnancy Loss in Women with HIV is not Associated with HIV Markers: Data from a National Study in Italy, 2001-2018	Floridia	2019	Italy	To explore risk factors for pregnancy loss.	Observational study; outcome: pregnancy loss and associated factors.	2696 pregnancies among WLHIV	Among 2696 eligible pregnancies reported between 2001 and 2018, 226 (8.4%) ended in pregnancy loss (miscarriage 198, 7.3%; stillbirth 28, 1.0%).<br>In multivariate analyses, only older age, HIV diagnosis before pregnancy and history of pregnancy loss were significantly associated with pregnancy loss.<br>No significant association with pregnancy loss was found for parity, coinfections, sexually transmitted diseases, hypertension, smoking, alcohol and substance use, CD4 cell count, HIV-RNA viral load, and CDC HIV stage.<br>Older women and those with a previous history of pregnancy loss should be considered at higher risk of pregnancy loss.
Prevalence, Correlates and Outcomes of Smoking in Pregnant Women with HIV: A National Observational Study in Italy	Floridia	2020	Italy	To assess smoking prevalence and its association with outcomes.	Multicentre observational study; outcomes included adverse pregnancy and neonatal outcomes.	3097 WLHIV	3097 pregnancies<br>The overall prevalence of reported smoking (at least one cigarette/day) was 25.6% (792/3097), with a significant decrease in recent years (19.0% in 2013–2018).<br>Women who smoked were less commonly African, had lower body mass index, older age, a longer history of HIV infection and higher CD4 counts.<br>In univariate analyses, smokers were significantly more likely to have PD, LB, FGR and detectable HIV viral load at third trimester.<br>Multivariable analyses confirmed for smokers a significantly higher risk of LB and FGR, while the associations with detectable HIV and PD were not maintained.<br>The common prevalence of smoking among pregnant women with HIV and its association with adverse outcomes indicates that smoking cessation programs in this population may have a significant impact on neonatal and maternal health.

CD4/CD8 ratio in pregnant women with HIV and its association with pregnancy outcome: data from a national study in Italy	Floridia	2021	Italy	To evaluate associations between CD4/CD8 ratio and pregnancy outcomes.	National study assessing CD4/CD8 ratio and associations with pregnancy outcomes.	934 pregnant WLHIV	Among 934 women, 536 (57.4%) were already on ART at conception. CD4/CD8 ratio (baseline value 0.570) increased significantly between the first and third trimesters, particularly in women who started ART in pregnancy. The rate of CD4/CD8 ratio normalqzation, defined by achieving a ratio ≥1 at the third trimester, was 13.2%.<br>In multivariable analyses, women who entered pregnancy with a CD4/CD8 ratio <0.3, compared to women with ratio ≥1, were almost four times less likely to have third-trimester HIV-RNA <50 copies/ml, and more than twice as likely to have preterm delivery.<br>For preterm delivery, a baseline CD4/CD8 ratio between 0.3 and 0.45 was also significantly associated with increased risk.<br>Described independent associations of low CD4/CD8 ratio with preterm delivery and HIVRNA suppression.
Incidence, patterns, and predictors of repeat pregnancies among HIV-Infected women in the United Kingdom and Ireland, 1990-2009	French	2012	United Kingdom Ireland	To investigate repeat pregnancies and associated characteristics.	Cohort study (NSHPC); outcomes included maternal and infant characteristics.	10568 WLHIV	14,096 pregnancies reported. Sub-analyses for predictors included 8,661 women with 11,426 pregnancies (first pregnancy during 2000–2009). 25.9% of women had repeat pregnancies.<br>The proportion of pregnancies in women who already had at least one pregnancy reported increased from 20.3% in 1997 to 38.6% in 2009.<br>Probability of repeat pregnancy significantly declined with increasing age at first pregnancy<br>Parity was also inversely associated with repeat pregnancy<br>Maternal health at first pregnancy was not associated with repeat pregnancy<br>The number of diagnosed HIV-infected women in the UK and Ireland experiencing repeat pregnancies is increasing. Variations in the probability of repeat pregnancies, according to demographic and clinical characteristics, are an important consideration
Presentation for care and antenatal management of HIV in the UK, 2009-2014	French	2017	United Kingdom	To assess timing of HIV care and adherence to clinical guidelines.	Observational study; outcomes included timing of HIV care and ART initiation.	5693 births from WLHIV	5693 births reported; 79.5% in women diagnosed with HIV prior to that pregnancy.<br>Median gestation at antenatal booking was 12.1 weeks and booking was significantly earlier during 2012–2014 vs. 2009–2011, although only in previously diagnosed women.<br>Overall, 42.2% of pregnancies were booked late.<br>Among women not already on treatment, antenatal ART commenced at a median of 21.4 weeks and started significantly earlier in the most recent time period.<br>Newly diagnosed women booked later for antenatal care and started antenatal ART later.<br>Multivariable analyses revealed demographic variations in access to or uptake of care, with migrants and parous women initiating care later.<br>Although women are accessing antenatal and HIV care earlier in pregnancy, some continue to face barriers to timely initiation of antenatal care and ART.

Pregnancy and neonatal outcomes among a cohort of HIV-infected women in a large Italian teaching hospital: a 30-year retrospective study.	Grignolo	2017	Italy	To examine long-term trends in pregnancy outcomes and associated risks.	Retrospective cohort (1985–2014); outcomes included preterm birth and infant HIV status.	231 WLHIV	262 deliveries<br>An increase in median age , in the proportion of foreigners, and a decrease in intravenous drug use in among pregnant WLHIV was observed.<br>HIV infections were diagnosed sooner (prior to pregnancy in 80% (56/70) of women in the last decade).<br>An increase in cART prescription during pregnancy and in HIV-RNA <50 copies/ml at delivery was observed.<br>The rate of elective caesarean section from 1985 to 1994 was 9·1%, which increased to 92·3% from 2004 to 2015.<br>Twelve (10·1%) MTCT occurred in the first decade, and six (8·3%) cases occurred in the second decade, the last of which was in 2000.<br>Preterm delivery (<37 weeks gestation) was 5% (6/121) from 1985 to 1994 and increased to 17·1% (12/70) from 2005 to 2014.<br>Advancing maternal age and previous pregnancies were associated with preterm delivery<br>Use of heroin or methadone was found to be the only risk factor for severe SGA.<br>Since 2000, MTCT has decreased to zero.
Not Recommended, But Done: Breastfeeding with HIV in Germany	Haberl	2021	Germany	To collect data on breastfeeding practices in women living with HIV in Germany.	Multicentre observational study (HELENE); outcomes included breastfeeding practices and maternal viral load.	42 WLHIV	64% born abroad (mostly sub-Saharan Africa).<br>Median time since HIV diagnosis 5 years.<br>CDC stage A (93%), stage B (7%).<br>Median BF duration 20 weeks.<br>All except one elite controller received ART<br>Viral load generally undetectable at delivery and during BF; two women had transient viral rebounds and stopped BF.<br>58% practiced exclusive BF, 42% mixed feeding.<br>Neo-PEP median 2 weeks; no adverse events in infants.<br>Increasing trend of BF WLHIV observed over time (1 case in 2009, 13 in 2018).<br>Highlights need for detailed guidelines and prospective data collection for safe BF in WLWH.
Reproductive desire in women with HIV infection in Spain, associated factors and motivations: a mixed-method study	Hernando	2014	Spain	To evaluate reproductive desire and associated factors in Spain.	Cross-sectional mixed-methods study; outcomes included reproductive desire and associated factors.	134 WLHIV	49% reported reproductive desire.<br>Desire was higher among women under 30, those without children, immigrants, and women not on antiretroviral treatment.<br>86.6% had previous pregnancies; 65% occurred before HIV diagnosis.<br>Main reasons for wanting children: liking children, forming a family<br>Main reasons for not wanting children: HIV infection, older age, already having children.<br>49% had sought information about safe pregnancy.<br>87% had disclosed HIV status to family; 39% reported discrimination.<br>Social support scores were generally high.

Reproductive history before and after HIV diagnosis: A cross-sectional study in HIV-positive women in Spain.	Hernando	2017	Spain	To examine reproductive history before and after HIV diagnosis.	Cross-sectional cohort study; outcomes included pregnancy outcomes and transmission rates.	161 WLHIV	Women aged 18–49 from 10 centres in 8 Spanish cities.<br>Of these, 138 women had been pregnant at least once, giving a total of 347 pregnancies<br>63 women had pregnancies after HIV diagnosis (101 pregnancies).<br>86% of women had been pregnant at least once; 39% had pregnancies after HIV diagnosis.<br>Of 101 post-diagnosis pregnancies, 20% were miscarriages and 29% were voluntary terminations.<br>Caesarean delivery increased from 20% pre-diagnosis to 51.8% post-diagnosis.<br>Vertical transmission occurred in 3 of 56 children born to HIV-positive mothers (5.4%), all in cases where HIV was diagnosed during delivery and ART was not received.<br>Pregnancy after HIV diagnosis was more likely in younger women at diagnosis, those diagnosed for ≥5 years, those who received antiretroviral therapy, and those who received reproductive health information.
Prenatal ultrasound screening and pregnancy outcomes in HIV-positive women in Germany: results from a retrospective single-center study at the Charite – Universitatsmedizin Berlin	Hofacker	2024	Germany	To assess mother-to-child transmission rates and prenatal screening outcomes.	Retrospective study; outcomes included delivery mode, complications, and neonatal outcomes.	420 WLHIV	420 pregnant WLHIV delivered 428 newborns<br>415 (98.8%) were receiving antiretroviral therapy (ART) and 88.8% had a viral load of <50 cop/ml prior to delivery.<br>46 (11%) of the newborns were born prematurely.<br>Low birth weight <2500 g occurred in 38 (9.1%) of the children.<br>219 (52.1%) caesarean sections were performed, most frequently due to previous CS (70.2%).<br>Eight severe malformations were detected using first and second trimester ultrasound.<br>One child had MTCT postpartum, resulting in an HIV transmission rate of 0.2%.<br>First and second trimester ultrasound screening is recommended, and reduction of unnecessary elective CS is advised to reduce complications in subsequent pregnancies.
Exploring the Intersection of Mental and Reproductive Health Among Women Living with HIV in Spain: A Qualitative Secondary Data Analysis	Huertas-Zurriaga	2025	Spain	To explore links between mental health and reproductive decision-making.	Secondary qualitative data analysis; thematic analysis applied.	26 WLWH	4 main themes were identified:<br>Emotional Fragmentation and Reproductive Self-Perception after the HIV Diagnosis<br>Coping Strategies in Reproductive Decision Making<br>Impacting Emotional Resilience and Motherhood Decisions<br>Emotions, Mental Health, and Desire for Motherhood<br><br>The themes revealed the profound emotional impact of HIV on their identity, mental health, and reproductive decision-making, affecting motherhood aspirations.

Predictors of pregnancy and changes in pregnancy incidence among HIV-positive women accessing HIV clinical care	Hunting ton	2013	United Kingdom	To describe predictors of pregnancy among women in HIV care.	Linked cohort data analysis; outcome: pregnancy incidence.	7853 WLHIV	The number of women accessing care at UK CHIC sites increased, as did the number of pregnancies.<br>Older women were less likely to have a pregnancy.<br>Women with CD4 cell count <200 cells/µL were less likely to have a pregnancy than those with CD4 200–350 cells/µL.<br>Women of white ethnicity were less likely to become pregnant compared with women of black African ethnicity.<br>The likelihood of pregnancy increased over the study period<br><br>Conclusion: The pregnancy rate among women accessing HIV clinical care increased in 2000–2009. HIV-positive women with, or planning, a pregnancy require a high level of care, and this need is likely to continue and increase as more women of older age conceive.
The risk of viral rebound in the year after delivery in women remaining on antiretroviral therapy	Hunting ton	2015	United Kingdom	To assess risk of viral rebound postpartum.	Cohort study comparing postpartum viral rebound with controls.	2732 WLHIV<br>618 WLHIV who conceived on cART + 1225 controls<br>321 WLHIV who started cART during pregnancy + 568 controls	In postpartum women who conceived on cART, 5.9% experienced viral rebound by 3 months, compared with 2.2% of their controls.<br>The risk of viral rebound was higher in postpartum women than in controls during the first 3 months [adjusted hazard ratio (aHR) 2.63, 95% CI 1.58–4.39], but not during the 3–12 months postdelivery/pseudo-delivery.<br>In postpartum women who started cART during pregnancy, 27% experienced viral rebound by 3 months, compared with 3.0% (1.6–4.4%) of their controls.<br>The risk of viral rebound was higher in postpartum women than in controls during both postdelivery/pseudo-delivery periods: <3 months, aHR 6.63 ; 3–12 months, aHR 4.05 .<br>In women on suppressive cART, the risk of viral rebound increases following delivery, particularly in the first 3 months, likely due to reduced adherence, highlighting the need for additional adherence support for postpartum women.
HIV Infection in Pregnancy and Adverse Perinatal Outcomes: A 14-Year Population-Based Study from a Central European Country Examining Preterm Birth, Low Birth	Hurt	2026	Czech Republic	To evaluate associations between maternal HIV and adverse perinatal outcomes.	Registry-based observational study; outcomes included low birth weight, SGA, and preterm birth.	164HIV-positive mothers<br>20,000 HIV-negative mothers (control)	Maternal HIV infection was associated with significantly increased odds of LBW (aOR 1.766, 95% CI 1.050–2.967; p = 0.032) and SGA (aOR 2.206, 95% CI 1.472–3.304; p < 0.001). The association with PTB was not statistically significant (aOR 1.251, 95% CI 0.693–2.259; p = 0.457). Infants born to HIV-positive mothers had lower mean birthweight and lower gestational age at delivery compared with HIV-negative controls. Caesarean delivery was more frequent among HIV-positive women (40.2 vs. 23.7%).<br>In this 14-year national cohort, maternal HIV infection was associated with impaired fetal growth—reflected by higher risks of LBW and SGA—but not with preterm birth. These findings underscore the need for enhanced fetal monitoring in pregnancies affected by HIV and support further investigation into the roles of ART regimens, maternal immune status, and regional epidemiological factors.

Weight, and Small-for-Gestational-Age Newborns							
Experience of the national cohort of pregnant women with HIV and their children in Spain: temporal trends in vertical transmission of HIV and associated infections.	Illan-Ramos	2024	Spain	To describe maternal and infant characteristics and outcomes.	Multicentre prospective study; outcomes included maternal and neonatal outcomes.	414 mother-child dyads (402 pregnant WLHIV, including 12 twin pregnancies).	Most mothers were immigrants (227/349; 65.1%).<br>Main route of HIV infection was heterosexual transmission, followed by vertical transmission<br>The diagnosis was made before conception in 313/389 women<br>394/402 (98%) received ART during pregnancy<br>356/402 (89.3%) an undetectable viral load at the time of delivery.<br>Delivery was vaginal in 230/388 children<br>Proportion of preterm birth was 11.1%<br>3 cases of vertical transmission of HIV<br>Only one newborn was breastfed.<br>most women living with HIV in Spain receive the diagnosis before conception, are of foreign ancestry and achieve good control of the infection. Although the VTR is very low in Spain, there are still infections that could be prevented with early diagnosis and treatment.
Perinatal outcomes of twenty-five human immunodeficiency virus-infected pregnant women: Hacettepe University experience	Inkaya	2020	Turkey	To assess perinatal outcomes in Turkey.	Retrospective study; outcomes included maternal clinical and neonatal characteristics.	25 WLHIV	26 singleton pregnancies from 25 WLHIV<br>Ethnicities were Turkish (n=18), East European (n=4), Asian (n=2) and African (n=2<br>Majority (76.9%) was aware of their HIV status before becoming pregnant.<br>4 cases (15.3%) were diagnosed during pregnancy and two (7.8%) at the onset of labor.<br>The results for median HIV viral load, CD4 count, and CD4/CD8 ratio at birth were 20 copies/mL 577/mm3, and 0.7 , respectively.<br>The rates of gestational diabetes mellitus, gestational hypertension, intrauterine growth restriction, and preterm delivery were 3.8%, 3.8%, 7.6%, and 8%, respectively.<br>Mean gestational week at birth was 38 weeks and mean birthweight is 2972±329 g.<br>Two babies were congenitally infected with HIV (infection rate of 8.3%).

Maternal characteristics during pregnancy and risk factors for positive HIV RNA at delivery: a single-cohort observational study (Brescia, Northern Italy)	Izzo	2011	Italy	To examine factors associated with detectable viral load at delivery among migrant women.	Observational study; outcome: detectable viral load at delivery and associated factors.	154 WLHIV	154 women (46.8% migrants) were included.<br>Presentation was later in migrant women than Italians, as assessed by CD4+ T-cell count at first contact<br>HIV diagnosis was made before pregnancy and HAART was already prescribed at the time of pregnancy in more Italians (91% and 75%, respectively) than migrants (61% and 42.8%, respectively).<br>A subgroup of women with available HIV RNA close to term was studied for risk factors of detectable HIV RNA at delivery.<br>Among 93 women, 25 had detectable HIV RNA.<br>A trend toward an association between non-Italian nationality and detectable HIV RNA at delivery was demonstrated by univariate analysis.<br>However, multivariable regression analysis showed the following factors to be more important: lack of stable (≥14 days) antiretroviral therapy at the time of HIV RNA testing and higher CD4+ T-cell count at pregnancy
Non-disclosure of a pregnant woman's HIV status to her partner is associated with non-optimal prevention of mother-to-child transmission.	Jassero n	2013	France	To study non-disclosure of HIV status and its implications for transmission prevention.	Cohort study (France); outcomes included HIV disclosure and PMTCT practices.	2952 WLHIV	15% of the women did not disclose their HIV status to their partner.<br>Non-disclosure was more frequent in women diagnosed with HIV infection late in pregnancy, originating from Sub-Saharan Africa or living alone, as well as when the partner was not tested for HIV.<br>Non-disclosure was independently associated with non optimal PMTCT<br>Rate of transmission did not differ according to disclosure status.<br>Factors associated with non-disclosure reflect vulnerability and its association with non optimal PMTCT is a cause for concern, although the impact on transmission was limited in this context of universal free access to care.
How women living with HIV in the UK manage infant-feeding decisions and vertical transmission risk - a qualitative study	Kasadh a	2024	United Kingdom	To explore infant-feeding decision-making and healthcare interactions.	Qualitative interview study; thematic analysis.	36 WLHIV	8 were pregnant and 28 postpartum.<br>The majority were of Black African descent (n=22) and born outside the UK.<br>The majority of postpartum women had an undetectable HIV viral load at the time of birth<br>The majority of postpartum women had chosen to formula feed. They were mainly motivated by removing all risk of HIV transmission (especially the two women who had not achieved virological suppression at the time of birth).<br>The minority of postpartum participants who had wanted to breastfeed (n=8) were mainly motivated by the health benefits of breastmilk to their baby (and one to herself and baby.<br>Those who had successfully breastfed also spoke of the bonding they experienced with their infant through breastfeeding, as well as breastfeeding allowing them to conform to personal, societal and cultural expectations of motherhood<br>A few women stopped breastfeeding earlier than planned due to a combination of a lack of tailored lactation support, unclear guidance, issues with breastmilk supply and/or cracked nipples<br>Women's decision making regarding infant-feeding was determined by (1)

							information and support; (2) practicalities of implementing medical guidance; (3) social implications of infant-feeding decisions
We decided together : a qualitative study about women with HIV navigating infant-feeding decisions with the father of their children	Kasadha	2024	United Kingdom	To investigate the role of fathers in infant-feeding decisions.	Qualitative interviews (NVivo analysis); outcomes: experiences of pregnancy and care.	36 WLHIV and 2 partners	Of the 36 women interviewed, 28 were postpartum.<br>The majority were of Black African descent (n = 22) and born outside the UK.<br>Key factors influencing how women navigated HIV and infant-feeding discussions with their baby's father included his: (1) awareness of the woman's HIV status, (2) relationship with the woman, (3) confidence in infant-feeding decisions, and (4) support for and opinion about the woman's infant-feeding intentions.<br>Most women in long-term relationships (>1 year) made joint decisions with the biological father.<br>Single women generally did not discuss their infant-feeding decisions with the father, often for safety reasons.<br>Fathers' awareness of the woman's HIV status, relationship quality, and confidence in the infant-feeding decision influenced involvement.
Negotiation of risk in sexual relationships and reproductive decision-making amongst HIV sero-different couples.	Kelly	2011	Northern Ireland	To understand reproductive decision-making in serodifferent couples.	Qualitative study using purposive sampling; outcomes: experiences of HIV diagnosis.	5 WLHIV, 1 HIV negative woman and 4 MLHIV	Among women, 2 were aware of their HIV diagnosis prior to pregnancy, 3 learned of their diagnosis in antenatal screening and<br>1 HIV-negative women choosing to conceive with an HIV-positive partner.<br>4 HIV-positive men were in sero-same or sero-different relationships, whose partner was actively trying to conceive, was pregnant or had recently given birth.<br>4 of the 6 women were born in Ireland, 1 of whom was with a partner born in Africa. The other 2 women were born in Africa and Eastern Europe. All 4 men were born in Africa but were now either working and/or studying in Northern Ireland.<br>Findings show that physical pleasure, love, commitment, a desire to conceive without medical interventions and a dislike of condoms within regular ongoing relationships shaped individuals' sense of biological risk. In addition, the subjective logic that a partner had not previously become infected through unprotected sex prior to knowledge of HIV status and the added security of an undetectable viral load significantly impacted upon women's and, especially, men's decisions to have unprotected sex in order to conceive. The findings speak to the importance of reframing public health campaigns and clinical counselling discourses on HIV risk transmission to acknowledge how couples negotiate this risk, alongside pleasure and commitment within ongoing relationships.

‘Every pregnant woman needs a midwife’—The experiences of HIV affected women in maternity care	Kelly	2013	Northern Ireland	To explore WLHIV experiences of pregnancy and maternity care, with a focus on their interactions with midwives.	Data collected through 22 interviews with 10 WLHIV, at different stage of their pregnancy A prospective repeat interview model was used, with interview schedules chosen to mirror the period of transition from reproductive decision-making through to parenthood.	10 WLHIV	Positive aspects: Knowledge and experience of HIV: Evaluation of whether their experience of care was positive or negative was often related to their perception of the midwife’s level of previous experience and knowledge of HIV.<br>Empathy: Highlighted the importance of being treated as ‘normal’, which invariably meant midwives who, regardless of their level of previous experience of HIV, focused on the ordinary aspects of pregnancy and did not allow the presence of HIV to overshadow their experience.<br>Continuity of care: Women appreciated the continuity of care provided by having a ‘named’ midwife.<br>Negative aspects: Conflicting information and a lack of communication, even among and between ‘wise staff’ in the specialist HIV and maternity unit, also led to a loss of confidence.<br>Breaches of confidentiality, by individuals and systems, lack of knowledge and experience, and the experience or perception of being treated differently because of their HIV status were central to participant’s negative experiences of care.<br>Continuing experiences and/or perceptions of people living with HIV that health-care professionals discriminated against them on the basis of their HIV status.
'If she gave birth to a healthy child, then she may forget about her own health': Postpartum engagement in HIV care and treatment among women living with HIV in Russia	King	2019	Russia	To identify factors influencing engagement in HIV care postpartum.	Qualitative interviews exploring barriers and facilitators to postpartum HIV care.	50 WLHIV and 20 service providers	Themes in regard to factors influencing postpartum engagement in care: feeling overwhelmed with caring for an infant; sense of responsibility for child’s health and well-being; misinformation and AIDS denialism; HIV-related stigma and fear; benefits versus side-effects of ART; professional and personal support; drug and alcohol use; and structural factors. Structural factors include poverty, continued provision of ART after pregnancy, dosage, obtaining necessary documents, and distance to clinic.
Social support and postpartum adherence to HIV treatment: a community-based participatory research study in Russia	King	2021	Russia	To assess social support and adherence to antiretroviral therapy postpartum.	Community-based survey; outcome: ART adherence postpartum.	200 WLHIV	40% of mothers reported adherence to ART<br>Supportive family environment positively associated with postpartum adherence<br>Active engagement with other HIV-positive mothers positively associated with adherence<br>Mothers with more than one child were less likely to be adherent<br>Support from relatives, partner support, childcare availability, household awareness of ART regimen and ease of asking for help were positively associated in bivariate analyses; wealth (above poverty) positively associated with adherence in bivariate analysis

Testing HIV positive in pregnancy: A phenomenological study of women's experiences.	Lingen-Stallard	2016	England	To explore women's experiences of receiving a positive HIV test result following antenatal screening, and to understand the personal experiences and emergent phenomenon of women testing HIV positive in pregnancy within an antenatal testing programme in England.	Qualitative phenomenological approach using individual, face-to-face, in-depth semi-structured interviews. Data analysed using reflective thematic analysis (holistic, selective, and detailed approaches).	13 WLHIV	13 Black African WLHIV<br>The emergent phenomenon was a transition and transformation of 'being' as women came to accept HIV as part of their lives.<br>Paired themes supported this process: shock and disbelief; anger and turmoil; stigma and confidentiality issues; acceptance and resilience.<br>Women described intense reactions to an HIV-positive diagnosis, compounded by cultural beliefs about imminent death.<br>Initial disbelief at the unexpected result evolved into sadness linked to the perceived loss of their former self.<br>Turmoil was evident as some women considered termination of pregnancy or experienced thoughts of self-harm.<br>Women often felt isolated, and relationship breakdowns were common.<br>Most reported pervasive stigma and described strategies to manage it while living with HIV.<br>Coping strategies included keeping HIV a 'secret' and making their child(ren) the central focus of their lives.<br>Resilience tended to increase over time.
Pregnancy outcomes in HIV-infected women of advanced maternal age	Liuzzi	2013	Italy	To assess advanced maternal age as a risk factor for adverse outcomes.	Observational study; outcomes included caesarean section, preterm birth, and birthweight.	1,578 WLHIV	1,578 pregnancies were analysed; 1,375 ended in live births, including 29 twin pregnancies and 1 triple pregnancy (total 1,406 newborns).<br>Median maternal age was 33 years.<br>Among 1,375 live births, 82.4% of deliveries were elective cesarean sections, 15.8% nonelective cesarean sections, and 1.8% vaginal deliveries.<br>Rates of nonelective cesarean section were similar among mothers ≥35 and <35 years.<br>Preterm delivery and low birthweight were more common among women ≥35 years in univariate but not multivariate analyses.<br>Overall birth defects rate was 3.4% with no differences by age. Older women were at higher risk of some adverse pregnancy outcomes, but associations did not persist in multivariate analyses.

HIV control in postpartum mothers: a turbulent time	Loftus	2016	United Kingdom	To audit postpartum management of HIV-positive women.	Retrospective case review; outcomes included postpartum care and follow-up.	76 WLHIV	A 84 pregnancies involving<br>mong women with previous AIDS-defining conditions or CD4 <350 cells/µL, 83% were correctly continued on ART.<br>Psychological or social problems were noted in 74% (62/84) of pregnancies.<br>Common issues included: depression (21%), partner-related concerns (21%), childcare problems (18%), financial difficulties (15%), housing problems (13%), and asylum issues (10%).<br>There was a statistically significant association between signs of depression and non-adherence.<br>All of the women who had poor virological control or stopped ART against medical advice had social issues or self-reported depression.<br>Of the women with a detectable VL, 86% (12/14) had psychological or social problems and 29% (4/14) showed signs of depression.<br>Non-attendance of appointments was often linked to psychological or social challenges.<br>Of the seven women who did not adhere to their postpartum management plan, all had psychological or social problems and 71% (5/7) self-reported depression.<br>Only 84.8% of women on ART maintained VL <200 copies/mL throughout the 12-month postpartum period, slightly below national averages
Pregnancy Outcomes Among Perinatally HIV-Infected Women in Spain	Lopez	2022	Spain	To assess clinical and virological outcomes in women with perinatally acquired HIV.	Multicentre retrospective study; outcomes included virological and pregnancy outcomes.	33 WLHIV	63 live births<br>At first delivery, median age was 20 years; 11 (33.3%) previously diagnosed with AIDS; 6 (18%) had mental health disorders.<br>40% became pregnant unsuppressed; 81% achieved viral suppression at delivery.<br>Treatment interruptions and losses to follow-up were common after delivery, with no positive effect of pregnancy on retention to care or immune virological status.<br>5 women (15%) experienced a new AIDS event; 2 deaths (6%) occurred during follow-up.<br>One case of MTCT occurred in a nonadherent woman where preventive measures could not be implemented.<br>Pregnancy poses particular challenges; multidisciplinary strategies are needed to minimise perinatal transmission risks and improve postpartum outcomes.

Trends in preterm birth in women living with HIV in Switzerland over the last three decades: A multicentric, prospective, cohort study	Lumbreras Areta	2024	Switzerland	To evaluate changes in preterm birth rates and associated factors in women living with HIV in Switzerland.	Cohort analysis (1986–2020); outcomes included preterm birth and associations with clinical and sociodemographic variables.	948 WLHIV	1238 pregnancies<br>The rate of PTB in WLHIV was highest prior to 2010 and progressively decreased thereafter, but remained higher than in the general population.<br>Older maternal age, lower CD4 count, detectable viraemia in the third trimester (T3), drug use, and mode of delivery were all significantly associated with PTB and period of study in univariate analysis.<br>There was no association between PTB and type of antiretroviral regimen.<br>No difference was observed in the rate of spontaneous labour between PTB and term delivery groups.<br>In multivariate analysis, only higher CD4 count at T3 and vaginal delivery were significantly associated with a decrease in PTB over time.
Management and outcomes of pregnancies among women with HIV in Oxford, UK, in 2008-2012	Montgomery-Taylor	2015	United Kingdom	To evaluate management and outcomes of pregnancies among women with HIV.	Retrospective cohort study; outcomes included viral load, delivery, and neonatal outcomes.	56 WLHIV	61 pregnancies<br>One case of MTCT (2%).<br>High viral load at delivery associated with preterm birth and low birth weight.<br>Most women delivered after 37 weeks; 6 preterm births (10%), 9 low birth weight (15%), 11 SGA (18%), 1 stillbirth (2%).<br>74% delivered by cesarean.<br>Most neonates received zidovudine prophylaxis; postnatal complications were uncommon.<br>High viral loads at booking or delivery linked to poorer neonatal outcomes.
The experience of pregnancy among women living with HIV in Nordic countries: A qualitative narrative enquiry	Moseholm	2022	Denmark Finland Sweden	To explore pregnancy experiences and social support in Nordic countries.	Qualitative narrative interview study.	31 WLHIV	In total, 31 women living with HIV were enrolled, of whom 61% originated from an African country and 29% from a Nordic country.<br>The analysis generated four primary narrative themes: just a normal pregnancy, unique considerations and concerns, interactions with healthcare, and social support.<br>Women living with HIV expressed a strong desire to have normal pregnancies and to be treated like any other pregnant woman.<br>However, this sense of normality was fragile, and pregnancy while living with HIV involved unique considerations and concerns, such as fear of transmission, antiretroviral therapy, and the need for specialised care, which were central to the women's experiences.<br>Interactions with healthcare providers and social support influenced their experiences in both positive and negative ways.
Infant feeding knowledge among women living with HIV and their interaction with healthcare providers in a high-income setting: a	Moseholm	2024	Denmark Finland Sweden	To assess knowledge and perceptions of infant feeding.	Longitudinal mixed-methods study (PACIFY); outcomes included infant feeding attitudes.	44 WLHIV	The merged analyses identified two overarching domains: Knowledge about breastfeeding in the U=U era and Communications with healthcare providers. The women expressed confusion about breastfeeding in the context of undetectable equals untransmittable (U=U). Women of Nordic origin were more unsure about whether breastfeeding was possible in the context of U=U than women of non-Nordic origin. Increased postpartum monitoring with monthly testing of the mother was not seen as a barrier to breastfeeding, but concerns were found regarding infant testing and infant ART exposure. Infant feeding discussions with healthcare providers

longitudinal mixed methods study.							were welcome but could also question whether breastfeeding was feasible, and many participants highlighted a need for more information.
Perception and Emotional Experiences of Infant Feeding Among Women Living With HIV in a High-Income Setting: A Longitudinal Mixed Methods Study	Moseholm	2024	Denmark Finland Sweden	To explore attitudes and experiences related to breastfeeding.	Mixed-methods cohort study; outcomes included breastfeeding perceptions.	44 WLHIV	44 women completed the survey, of whom 31 also participated in qualitative interviews.<br>All participating women identified as cisgender.<br>12 women were born in a Nordic country while 32 were born outside of the Nordic countries; 24 of whom were of African origin while 8 originated from South America, Southern or Eastern Europe, or the Middle East<br>3 overarching domains representing commonalities across the quantitative and qualitative data: emotional impact, justifying not breastfeeding, and coping strategies.<br>Not being able to breastfeed was emotionally challenging.<br>Cultural expectations influenced the women's experiences and the strategies they used to justify their infant feeding choice.
Psychosocial health in pregnancy and postpartum among women living with-and without HIV and non-pregnant women living with HIV living in the Nordic countries - Results from a longitudinal survey study	Moseholm	2022	Denmark Finland Sweden	To assess psychosocial health outcomes and depression risk.	Longitudinal survey study; outcomes included depression, stress, and social support.	47 WLHIV	The prevalence of probable depression among pregnant WLHIV was 24% in the third trimester, 36% at 3 months postpartum, and 22% at 6 months postpartum.<br>There was no difference in depression scores or perceived stress scores between pregnant and non-pregnant WLHIV at any time point.<br>Pregnant WLHIV reported significantly higher depression scores and more perceived stress at all time points compared to pregnant WWOH.<br>Pregnant WWOH reported significantly less stress over time while there was no change in perceived stress in any of the WLHIV groups.<br>Pregnant WLHIV reported significantly lower scores on the UCLA loneliness scale at T1 compared to non-pregnant WLHUV and significantly higher loneliness scores at T1 compared to pregnant WWOH. However, over time there was a significant increase in loneliness scores among pregnant WLHIV.<br>There was no significant difference in loneliness scores between pregnant and non-pregnant WLHIV, and between pregnant WWH and pregnant WWOH at T2 and T3.<br>Pregnant and non-pregnant WLHIV reported similar MSPSS social support scores over time and there was no significant difference within or between groups. Compared to the pregnant WWOH, pregnant WLHIV at all times and in all sub-scales reported significantly less social support.<br>Only loneliness score in pregnancy remained significantly associated with increased odds of probable depression in pregnancy while social support in pregnancy was associated with a decreased odds of probable depression in pregnancy .<br>Only postpartum loneliness remained significantly associated with probable postpartum depression in the adjusted analysis*<br><br>High burden of adverse psychosocial outcomes was observed in both

							pregnant and non-pregnant women living with HIV compared to pregnant women without HIV. Loneliness and inadequate social support were associated with increased odds of depression in pregnancy and should be a focus in future support interventions.
Retention in care and viral suppression in pregnant/postpartum vs. nonpregnant/nonpostpartum women with HIV	Moseholm	2025	Denmark	To investigate retention in care and virological outcomes.	Registry linkage study; outcomes included retention in care and viral suppression.	564 pregnant WLHIV and 1705 nonpregnant/nonpostpartum WLHIV	Women aged 15–50 years.<br>169 women had more than one pregnancy during study.<br>Retention in care was lower during pregnancy and in the second postpartum year compared to nonpregnant women.<br>Viral suppression was comparable overall, though slightly lower in the second postpartum year.<br>Virological failure was higher in pregnant women but lower in postpartum women, particularly in the second postpartum year.<br>ART initiation during pregnancy and diagnosis during pregnancy were associated with adverse outcomes; more than 5 years since diagnosis improved retention.<br>Maternal age, ART timing, and socioeconomic factors influenced outcomes.<br>Maternal psychiatric illness was not associated with adverse outcomes.<br>Overall, recent deliveries linked to better HIV care outcomes.
Induced abortions of women living with HIV in Finland 1987-2019: a national register study	Mutru	2023	Finland	To determine rates and factors associated with induced abortion among women living with HIV in Finland.	Nationwide retrospective register study; outcomes included induced abortion rates and associated factors (multivariable regression).	1017 WLHIV	Rate of induced abortions among WLHIV decreased from 42.8 to 14.7 abortions per 1,000 follow-up years from 1987–1997 to 2009–2019, more prominently in abortions after HIV diagnosis.<br>After 1997, being diagnosed with HIV was not associated with an increased risk of terminating a pregnancy.<br>Factors associated with induced abortion in pregnancies that began after HIV diagnosis (1998–2019) were being foreign-born, younger age, previous induced abortions, and previous deliveries.<br>Estimated prevalence of undiagnosed HIV at induced abortion was 0.008–0.029%.<br>Rate of induced abortions among WLHIV has decreased.

Child desire in women and men living with HIV attending HIV outpatient clinics: Evidence from a European multicentre study	Nöstlinger	2013	Austria Belgium Czech Republic Germany Greece Hungary Italy Latvia Poland Portugal Slovakia Spain United Kingdom	To identify factors influencing desire for children.	Cross-sectional survey; outcome: fertility intentions.	229 women and 197 men living with HIV. 1 gender missing	43% reported a child desire, 28% had unmet family planning needs. Factors independently associated with child desire were: being younger than 36 years for both genders, and having no children for women. Perceived HIV-related discrimination by health care providers was associated negatively with child desire for women, but positively for men Many women and men living with HIV in Europe desire children and have fertility intentions
Over a third of childbearing women with HIV would like to breastfeed: A UK survey of women living with HIV	Nyatsanza	2021	United Kingdom	To explore attitudes towards breastfeeding in the UK.	Questionnaire study; outcomes included breastfeeding attitudes.	94 WLHIV, pregnant or within 3 months post-partum	69% Black African<br>74% immigrants<br>Median age 36, median CD4 count 618 cells/mm³, 92% had undetectable viral load.<br>38% wanted to breastfeed despite HIV, 89% would if HIV-negative.<br>66% felt pressured to invent reasons for not breastfeeding<br>62% questioned by friends/family.<br>Half concerned about HIV transmission or ART side effects.<br>Knowledge gaps: only two-thirds knew it was unsafe with detectable viral load; understanding of safety with undetectable viral load inconsistent.<br>Cultural expectations and stigma influenced choices.
Association of pregnancy with engagement in HIV care among women with HIV in the UK: a cohort study.	Okhai	2021	United Kingdom	To examine engagement in HIV care before, during, and after pregnancy.	Cohort study; outcome: engagement in HIV care.	1116 matched pairs of pregnant and non-pregnant WLHIV	Among pregnant women, the proportion of time engaged in care increased during pregnancy and after pregnancy, compared with before pregnancy. Among non-pregnant women in the control group, engagement in HIV care remained stable across the three equivalent stages The association of engagement in HIV care with pregnancy or pseudo pregnancy stage differed significantly by case-control status the odds of engagement in HIV care were higher during pregnancy and after pregnancyonly among pregnant women, and not among non-pregnant women, when compared with the before pseudo pregnancy stage.<br>WLHIV and a pregnancy resulting in a live birth were more likely to engage in HIV care postpartum when compared with before pregnancy.

Changes in characteristics and HIV-clinical outcomes of pregnant people living with HIV in the UK	Okhai	2025	United Kingdom	To analyse demographic and clinical trends over time.	Linked cohort analysis; outcomes included CD4 changes and viral rebound.	4341 pregnant WLHIV	Maternal age increased from 31 to 34.<br>Those conceiving in the most recent period had been diagnosed with HIV for longer, had a higher median CD4 count, and median nadir CD4 count, they were also more likely to have initiated ART prior to estimated conception, and have a suppressed conception VL.<br>Median CD4 count change from delivery to 12 months post-partum showed no difference across periods; cumulative proportion with viral rebound at 12 months post-delivery decreased over time.<br>Clinical management of pregnant people has changed over time, resulting in positive trends in this study both within pregnancy and postpartum.
Assessment of mode of delivery and predictors of emergency caesarean section among women living with HIV in a matched-pair setting with women from the general population in Denmark, 2002-2014	Ørbæk	2017	Denmark	To assess predictors of emergency caesarean section.	Case–comparison study; outcomes included delivery mode and caesarean section indications.	389 WLHIV and 1945 WGP	At delivery, all WLHIV were on antiretroviral therapy and most had HIV RNA <40 copies/mL.<br>Mode of delivery differed significantly between WLHIV and women in the general population : vaginal delivery 33.4% vs 73.3%; elective caesarean section 40.6% vs 9.7%; emergency caesarean section 26% vs 17%.<br>No mother-to-child transmission occurred.<br>Increasing numbers of WLHIV deliver vaginally.<br>Despite virological suppression, more WLHIV plan and deliver by ECS than WGP.<br>WLHIV had a twofold higher risk of EmCS compared with WGP.
Viral suppression and retention in HIV care during the postpartum period among women living with HIV: a longitudinal multicenter cohort study	Paioni	2023	Switzerland	To investigate retention in care and infant follow-up postpartum.	Prospective cohort; outcomes included maternal and infant HIV outcomes.	586 WLHIV and 553 children	Included 737 deliveries in 586 mothers in the analysis.<br>553 children were included in the offspring outcome analysis after pooling siblings from twin (11) and triplet (1) deliveries into a single observation.<br>Overall, WLHIV were retained in HIV care for at least six months after 94.2% of deliveries (694/737).<br>Late initiation of cART during the third trimester was the main risk factor for failure to remain in care.<br>Among mothers on cART for at least one year post-delivery, 4.4% (26/591) experienced viral failure, with illicit drug use being the most significant risk factor<br>The main risk factor for non-adherence to infant follow-up recommendations was maternal depression.<br>Although overall outcomes are reassuring, several modifiable risk factors for adverse postpartum outcomes—such as late treatment initiation and depression—were identified.

Pregnancy outcomes of HIV-positive women in a tertiary centre in the UK	Pammi	2015	United Kingdom	To evaluate management of detectable viral load in late pregnancy.	Retrospective review; outcomes included ART management and viral load.	106 WLHIV	106 pregnancies<br>Average age at booking 29 years.<br>48 women diagnosed via antenatal screening and 58 previously diagnosed.<br>Majority from sub-Saharan Africa (91/106); others from Jamaica (2), Belgium (1), Thailand (1), UK (11).<br>23 women were ART-naive, 11 had prior HAART exposure in previous pregnancy, 24 were on HAART at booking.<br>20 women (19%) had detectable viral load at 36 weeks or before in preterm deliveries.<br>Of 20 women with detectable viral load, 11 were planned for CS (10 CS, 1 spontaneous vaginal delivery). Four women with low detectable viral load at 36 weeks delivered vaginally with intrapartum AZT after close monitoring.<br>Four women received treatment intensification with raltegravir; five received single-dose nevirapine before delivery.<br>Neonates born to women with detectable VL received 4-week triple prophylaxis.<br>One intrauterine HIV transmission occurred in a mother who seroconverted late and delivered outside hospital; all other infants remained HIV-negative. Overall mother-to-child transmission rate was <1%; 0% in women with undetectable viral load at 36 weeks.
National audit of perinatal HIV infections in the UK, 2006-2013: what lessons can be learnt?	Peters	2018	United Kingdom	To investigate circumstances of perinatal HIV transmission.	National surveillance study; outcomes included timing of diagnosis and transmission.	108 WLHIV mother-child pair	A total of 108 PHIVs were identified.<br>Nearly 90% (94 of 108) of the mothers were born abroad (mainly in Africa).<br>Of the 41 (38%) infants whose mothers were diagnosed before delivery, it is probable that most were infected in utero, around 20% intrapartum, and 20% through breastfeeding.<br>Timing of transmission was unknown for most children of undiagnosed mothers.<br>For infants born to diagnosed women, the most common contributing factors for transmission were difficulties with engagement and/or ART adherence in pregnancy (14 of 41) and late antenatal booking (nine of 41).<br>For the 67 children with undiagnosed mothers, the main factors were decline of HIV testing (28 of 67) and seroconversion (23 of 67).<br>Adverse social circumstances around the time of pregnancy were reported for 53% of women, including uncertain immigration status, housing problems, and intimate partner violence.<br>8 children died, all born to undiagnosed mothers.

Operative vaginal delivery and invasive procedures in pregnancy among women living with HIV	Peters	2017	United Kingdom Ireland	To describe operative delivery outcomes.	Surveillance study; outcomes included operative delivery and infant HIV status.	7,417 WLHIV	9,372 pregnancies (2008–2016) in 7,417 WLHIV women<br>Median maternal age: 33 years for operative deliveries, 38 years for invasive procedures.<br>3,663 vaginal deliveries, including 3023 wit data on instrument use.<br>Among 3,023 vaginal deliveries with data, 251 infants (8.2%) were delivered operatively, predominantly with forceps (67%) over vacuum (29%), increasing slightly over time.<br>For invasive procedures, 4,063 pregnancies (2012 onwards) with 2,163 reporting data.<br>Operative delivery more common in nulliparous women and term deliveries.<br>One HIV transmission reported in an operative delivery, with additional risk factors.<br>Among 27 invasive procedures, all but one woman were on cART with undetectable VL.<br>Procedures included 25 amniocenteses, one CVS, one cordocentesis.<br>Outcomes: 23 livebirths, 2 stillbirths, 2 terminations; no HIV transmissions reported in surviving infants.<br>Findings support the relative safety of operative deliveries and invasive procedures in women on cART, though numbers remain low and continued monitoring is recommended.
HIV and induced abortion among migrants from sub-Saharan Africa living in Ile-de-France: Results of the PARCOURS study	Pilecco	2024	France	To assess abortion rates among migrant women.	Cross-sectional survey (PARCOURS); outcome: induced abortion rates.	242 HIV negative women and 277 HIV positive women	Among the respondents, 77 in the reference (33.5 %), and 113 in the HIV group (39.5 %) declared having at least one induced abortion after arrival in France, without a statistically significant difference.<br>Pregnancies in the HIV group were more frequently terminated in abortion (14.1% vs. 11.0 %), occurred at older ages, were more frequently unwanted and less frequently intended, less often resulted from long-term relationships only, were more frequently preceded by previous abortions, and occurred within a shorter time since migration compared to pregnancies in the reference group.<br>In the reference group, 11.0 % of pregnancies were terminated in abortion, the same situation as 14.1 % in the HIV group (p = 0.124).<br>HIV was not associated with abortion in the crude and adjusted models. However, after adjustments, HIV exhibited a non-significant trend towards reducing the likelihood of abortion, particularly when considering the intendedness of pregnancy variable.<br>Factors that shape the overall context of women's lives and pregnancies, may have a more significant impact on reproductive decision-making than HIV alone. Health services must pay attention to the intendedness of pregnancies, providing advice and support on the prevention of MTCT to WLHIV who intend to become pregnant,

Factors Affecting Antiretroviral Therapy Adherence among HIV-Positive Pregnant Women in Greece: An Exploratory Study	Pontiki	2022	Greece	To evaluate adherence to antiretroviral therapy in pregnancy.	Questionnaire-based study; outcomes included social support and ART adherence.	200 pregnant WLHIV	Overall ART non-compliance: 13%<br>Greek nationality associated with higher compliance<br>Higher educational level associated with higher compliance<br>Higher knowledge scores associated with higher compliance<br>Low social support associated with lower compliance<br>Compliance influenced by individual, social, and health system factors<br>Compliance with ART in HIV-positive pregnant women in Greece is 13%, a rate almost 50% lower than that observed in developing countries.
Pregnancy outcomes in perinatally HIV-infected young women in Madrid, Spain: 2000-2015	Prieto	2017	Spain	To describe prevention strategies and infant outcomes.	Prospective cohort (Madrid); outcomes included infant HIV status and SGA.	22 WLHIV	28 pregnancies among PHIV women.<br>Most women were Caucasian and heavily treatment-experienced.<br>9 cases (32.1%) were at high risk for HIV MTCT.<br>TC-DRM were documented in 3 women, and salvage regimens led to undetectable VL close to delivery.<br>4 out of 6 PHIV women under virological failure after 28 weeks were not on ART before conception; despite early recognition,VL was not fully suppressed at week 28 in all cases.<br>One patient rejected ART and psychosocial support.<br>In 4 cases, a new ART drug was added including raltegravir, leading to undetectable VL close to delivery in two cases.<br>Overall, maternal VL was detectable close to delivery in 4 women (14.3%).<br>Elective cesarean section was performed in these cases, and infants received three-drug postnatal prophylaxis.<br>None of the newborns acquired HIV infection.<br>8 infants (28.6%) were small for gestational age.<br>The study highlighted management challenges in this population and the need for specific strategies to minimise perinatal transmission risks.
Does pregnancy affect the early response to cART?	Rachas	2013	France	To assess impact of pregnancy on treatment response.	Cohort analysis; outcomes included virological response to ART.	892 WLHIV	779 pregnant and 113 nonpregnant<br>Pregnancy did not affect early virological response to cART when threshold was <400 copies/ml at 1, 3, or 6 months<br>CD4 recovery was similar in pregnant and nonpregnant women.<br>Pregnant women were less likely to reach <50 copies/ml at 3 months, mostly due to shorter treatment duration before delivery.<br>By delivery, only 63.8% of pregnant women had <50 copies/ml.<br>Viral load decline ≥2 log_{10} at 1 month was similar between groups.<br>MTCT rate was 1.2% (9 infants).<br>Overall conclusion: Pregnancy does not biologically impair virological or CD4 response to cART; suboptimal suppression at delivery is primarily due to shorter duration of treatment.

The management of HIV in pregnancy: A 10-year experience	Raffe	2017	United Kingdom	To review HIV management in pregnancy and transmission rates.	Retrospective review; outcomes included HIV management and pregnancy outcomes.	75 WLHIV	97 pregnancies in 75 women were identified, resulting in 79 live births.<br>Antenatal HIV diagnosis was made in 22 (28%). The proportion of pregnancies in women with known HIV at conception increased over time.<br>At conception, 58 (60%) were on ART, of whom 33 (57%) continued their original regimen.<br>34 (35%) initiated ART following conception: 14 were known to be HIV positive and 20 were diagnosed during pregnancy.<br>2 did not start ART (1 due to miscarriage and 1 diagnosed post-delivery), and in 3 cases ART history was unavailable due to transfer to alternative centres.<br>Planned mode of delivery was documented in 73 cases: 30 (41%) planned a normal vaginal delivery and 43 (59%) a caesarean section.<br>Viral load was <50 copies/mL in 58 (76%) at 36 weeks and 64 (84%) at delivery. 90% of those with a detectable viral load at 36 weeks delivered by caesarean section.<br>All infants received neonatal post-exposure prophylaxis. 84% initiated prophylaxis within 4 hours of birth, and 90% completed 28 days.<br>Over the 10-year review period, 1 infant (1.3%) was diagnosed HIV positive.
Pregnancy complications in HIV-positive women: 11-year data from the Frankfurt HIV Cohort.	Reitter	2014	Germany	To assess pregnancy complications over time.	Observational cohort; outcomes included maternal and neonatal morbidity.	330 pregnancies in WLHIV	GDM was diagnosed in 38 of 330 women (11.4%).<br>5 women (1.5%) developed pre-eclampsia or hypertension.<br>In 16 women (4.8%), PROM occurred and 46 women (13.7%) were admitted with preterm contractions.<br>Preterm delivery rate was 36.5% (n = 122).<br>Over the observation period, the percentage of women with undetectable HIV viral load increased from 26.1% to 75%, leading to obstetric changes, including an increase in the rate of vaginal deliveries, from no vaginal births to 50%.<br>The preterm delivery rate decreased from 79.2% to 8.3%.<br>No significant changes in the rate of GDM, pre-eclampsia, PROM or preterm contractions.<br>The rates of complications such as GDM, pre-eclampsia, preterm contractions, PROM and postnatal complications were stable over the 11 years, but were still increased compared with the general population.

Antiretroviral therapy during pregnancy and premature birth: Analysis of Swiss data	Rudin	2011	Switzerland	To examine associations between antiretroviral therapy and prematurity.	Prospective cohort study; outcomes included prematurity and birthweight.	1040 WLHIV	1180 pregnancies<br>Odds ratios for prematurity in women receiving mono/dual therapy and cART were 1.8 and 2.5 compared with women not receiving ART during pregnancy. In a subgroup of 365 pregnancies with comprehensive information on maternal clinical, demographic and lifestyle characteristics, there was no indication that maternal viral load, age, ethnicity or history of injecting drug use affected prematurity rates associated with the use of cART.<br>Duration of cART before delivery was also not associated with duration of pregnancy.<br>Confounding by maternal risk factors or duration of cART exposure is not a likely explanation for the effects of ART on prematurity in HIV-1-infected women.
Unplanned pregnancies and social and partner support during pregnancy in Spanish women living with HIV	Ruiz-Alguero	2023	Spain	To describe unplanned pregnancies and social support.	Cross-sectional study; outcomes included reproductive health behaviours.	38 WLHIV	Median age at pregnancy was 36 years.<br>27 (71.1%) women were born outside of Spain, mainly in sub-Saharan Africa (39.5%), and 17 (44.7%) were employed.<br>34 (89.5%) women had previous pregnancies, and 32 (84.2%) had experienced previous abortions or miscarriages.<br>17 (44.7%) women had shared with their clinician their desire to become pregnant.<br>34 (89.5%) pregnancies were natural, and four used assisted reproductive techniques (in vitro fertilisation; one additionally used oocyte donation).<br>Among the 34 women with natural pregnancies, 21 (61.8%) were unplanned, and 25 (73.5%) had information on how to become pregnant while avoiding HIV transmission to the baby and partner.<br>Women who did not seek advice from their physician about becoming pregnant had a significantly increased risk of unplanned pregnancy.<br>Overall, 14 (36.8%) women reported low social support during pregnancy, and 27 (71.0%) reported good or very good support from their partner.
Previous reproductive history and post-natal family planning among HIV-infected women in Ukraine	Saxton	2010	Ukraine	To assess reproductive health and contraception use.	Prospective cohort study; outcomes included contraception use postpartum.	371 WLHIV	69% diagnosed with HIV during most recent pregnancy.<br>77% sexually active post-natally.<br>21% reported no contraception use (most not sexually active).<br>50% used condoms, 20% coitus interruptus, 4% hormonal or IUD.<br>Consistent condom use in pregnancy, HIV-discordant status, current sexual activity, and study site associated with post-natal condom use.<br>Affordability of contraception and inconsistent condom use in pregnancy associated with non-use of contraception<br>Effective post-natal contraception use was low.<br>HIV-positive partners less likely to use condoms post-natally.

Update of Perinatal Human Immunodeficiency Virus Type 1 Transmission in France: Zero Transmission for 5482 Mothers on Continuous Antiretroviral Therapy From Conception and With Undetectable Viral Load at Delivery	Sibiude	2023	France	To re-estimate preterm birth rates by treatment timing.	Cohort analysis (France); outcomes included preterm birth trends.	14 630 WLHIV (mother–infant pairs)	Preterm (PT) birth decreased across three periods: from 1.1% in 2000–2005 (58/5,123) to 0.7% in 2006–2010 (30/4,600) and 0.2% in 2011–2017 (10/4,907; P < 0.001).<br>Among the 6,316/14,630 (43%) women on ART at conception, PT decreased from 0.42% (6/1,434) in 2000–2005 to 0.03% (1/3,117) in 2011–2017 (P = 0.007).<br>For women treated at conception with undetectable maternal plasma viral load (pVL) near delivery, no PT was observed regardless of ART combination [(0/5,482).<br>Among women who started ART during pregnancy and had undetectable pVL near delivery, PT was 0.57%(26/4,596).<br>Among women treated at conception but with detectable pVL near delivery, PT was 1.08% (9/834).<br>Additionally, 10 cases of mother-to-child transmission were qualitatively described during 2011–2017.<br>In a setting with free access to ART, monthly pVL monitoring, infant ART prophylaxis, and no breastfeeding, suppressive ART initiated before pregnancy and continued throughout pregnancy can reduce preterm birth among women with HIV to almost zero.
Premature Delivery in HIV-Infected Women Starting Protease Inhibitor Therapy During Pregnancy: Role of the Ritonavir Boost?	Sibiude	2012	France	To assess associations between therapy and prematurity.	Cohort study; outcomes included prematurity and ART exposure.	1,253 WLHIV	Of the 1,253 women starting PI-based ARV during pregnancy, majority from sub-Saharan Africa, multiparous, ARV naive, started treatment during second trimester.<br>Prematurity increased from 9.2% (1990–1993, no therapy) to 14.3% (2005–2009, routine cARV).<br>Prematurity associated with cARV vs zidovudine monotherapy.<br>During 2005–2009, prematurity higher with boosted than nonboosted PI therapy started during pregnancy; Difference mainly due to induced preterm delivery for maternal or fetal indications.<br>Prematurity among HIV-infected pregnant women twice that in general population in France, independently associated with cARV and initiation of ritonavir-boosted PI therapy.
Diagnosis of HIV infection during pregnancy: Trends from a national cohort in Spain	Suarez Garcia	2025	Spain	To examine pregnancies diagnosed during pregnancy.	Cohort study; outcomes included pregnancies diagnosed during pregnancy.	185 WLHIV	Of 2,102 women, 185 (8.8%) were diagnosed with HIV during pregnancy. Among these, 51.2% were late presenters, while 25.4% and 9.2% were diagnosed during the second and third trimesters, respectively. Women from Latin America and Sub-Saharan Africa were more likely to be diagnosed after the first trimester compared to Spanish women.<br>Overall, 95.7% initiated ART during pregnancy. Over time, the use of emtricitabine plus tenofovir disoproxil fumarate (and later emtricitabine plus tenofovir alafenamide), as well as integrase strand transfer inhibitors, increased.<br>In total, 95.1% of pregnancies resulted in delivery, of which 46.0% were by caesarean section. At 36 weeks of pregnancy, 82.8% of women had an undetectable VL, increasing from 71.7% in 2004–2008 to over 95% after

							2013. Preterm birth and low birth weight occurred in 10.0% and 9.8% of deliveries, respectively, with one case of perinatal HIV transmission.
Pregnancy in Women with HIV: Incidence and Outcomes over 19 Years in a Multi-Center Cohort in Spain	Suarez Garcia	2025	Spain	To describe pregnancy trends in cohort studies.	Prospective cohort (CoRIS); outcomes included pregnancy outcomes and transmission.	2102 ART-naïve WLHIV	358 (17.0%) became pregnant over 15,586 women-years of follow-up (509 pregnancies in total).<br>Pregnancy incidence rate was 32.6 per 1000 women-years (95% confidence interval: 29.9, 35.6), which remained stable throughout the study. In 78.8% of the pregnancies, women were on ART at their last menstrual period, increasing from 49.2% in 2004 to 94.8% in 2022.<br>In 43.4% of pregnancies, women received ART regimens not recommended during pregnancy.<br>Treatment changes occurred in 43.1% of pregnancies, mainly in the first trimester (74.6%).<br>76.2% of pregnancies resulted in delivery, of which 48.2% were cesarean.<br>Spontaneous and medical abortions occurred in 12.5% and 10.5% of pregnancies, respectively.<br>In 92.0% of deliveries, women had an undetectable VL at 36 weeks. The proportion of cesarean births among those with undetectable VL was 45.9%.<br>Eighteen percent of newborns were preterm, and 11.5% had low birth weight.<br>There was one neonatal death and one HIV perinatal transmission.<br>Most pregnancies occurred in women on ART with undetectable VLs at delivery. Despite this, there was a high proportion of cesarean births.<br>Many women received ART regimens not recommended during pregnancy with nearly half changing their treatment at least once.
"It pains me because as a woman you have to breastfeed your baby": decision-making about infant feeding among African women living with HIV in the UK.	Tariq	2016	United Kingdom	To explore infant-feeding decisions among African women.	Qualitative study; outcomes included pregnancy experiences and stigma.	23 WLHIV	Women highlighted the cultural importance of breastfeeding in African communities and the social pressure to breastfeed, also describing fears that replacement feeding would reveal their HIV status. Participants expressed significant concerns about the physical and psychological effects of replacement feeding on their child and felt that their identity as good mothers was compromised by not breastfeeding. However, almost all chose to refrain from breastfeeding, driven by the desire to minimise vertical transmission risk. Participants' resilience was strengthened by financial assistance for replacement feeding, examples of healthy formula-fed children, and support from partners, family, peers, and professionals.

Loss to Follow-Up After Pregnancy Among Sub-Saharan Africa-Born Women Living With Human Immunodeficiency Virus in England, Wales and Northern Ireland: Results From a Large National Cohort	Tariq	2016	England Wales Northern Ireland	To assess loss to follow-up by demographic factors.	Retrospective cohort; outcome: loss to follow-up.	5390 WLHIV	7211 pregnancies<br>Overall, 12.5% of women did not return for HIV care in the year after pregnancy.<br>SSA-born women had higher odds of LTFU than white UK-born women.<br>SSA-born women who migrated during pregnancy had the highest odds of LTFU.<br>Younger maternal age, higher CD4 count, detectable viral load, and shorter duration of UK residence were also associated with higher LTFU.<br>No significant association with injecting drug use or pregnancy outcome.
Prevention of mother-to-child transmission of human immunodeficiency virus among pregnant women using injecting drugs in Ukraine, 2000-10	Thorne	2012	Ukraine	To compare outcomes between injecting drug users and non-users.	Prospective cohort; outcomes included MTCT and birth outcomes.	6200 WLHIV	Of 6,200 women, 1,111 (18%) reported current or previous IDU.<br>The proportion of IDUs diagnosed with HIV before conception increased from 31% in 2000/01 to 60% in 2008/09 .<br>Among women with undiagnosed HIV at conception, 20% of IDUs were diagnosed intrapartum versus 4% of non-IDUs.<br>At enrolment, 14% of IDUs had severe or advanced HIV symptoms versus 6% of non-IDUs.<br>IDUs had higher rates of PTD and LBW infants than non-IDUs: 16% versus 7% and 22% versus 10%, respectively.<br>IDUs were more likely to receive no neonatal or intrapartum PMTCT prophylaxis compared with non-IDUs.<br>MTCT rates were 10.8% in IDUs versus 5.9% in non-IDUs; IDUs had increased MTCT risk.<br>Fewer IDUs with treatment indications received HAART compared with non-IDUs.<br>Pregnant HIV IDU have worse clinical status, poorer access to prevention of MTCT prophylaxis and HART, more adverse pregnancy outcomes, and a higher risk of MTCT than non-injecting drug user women.
Vaginal delivery in women with HIV in Italy: results of 5 years of implementation of the national SIGO-HIV protocol	Tibaldi	2019	Italy	To evaluate safety of vaginal delivery protocols.	Multicentre observational study; outcomes included delivery outcomes and complications.	580 WLHIV	142 (24.5%) had vaginal delivery, 323 (55.7%) ECS, 115 (19.8%) NECS.<br>Vaginal delivery increased over time (18.9% in 2012 to 35.3% in 2017).<br>Women delivering vaginally were younger, more often nulliparous, diagnosed during current pregnancy, and antiretroviral-naive; had slightly longer gestation and higher birthweight.<br>NECS associated with more adverse outcomes and delivery complications.<br>HIV transmission rate was minimal (0.4%) with no vertical transmission in vaginal delivery group.

Maternal and foetal outcomes among 4118 women with HIV infection treated with lopinavir/ritonavir during pregnancy: analysis of population-based surveillance data from the national study of HIV in pregnancy and childhood in the United Kingdom and Ireland	Tookey	2016	United Kingdom Ireland	To assess outcomes of specific antiretroviral regimens.	Retrospective cohort; outcomes included ART exposure and neonatal outcomes.	4118 pregnant WLHIV	4864 pregnancies<br>Median maternal age 30 years<br>77% of women were of sub-Saharan African origin.<br>Viral suppression (<50 copies/mL) achieved in 73% at delivery.<br>63% of deliveries by caesarean.<br>Preterm births <37 weeks: 13%; low birth weight <2500 g: 15%.<br>MTCT decreased from 1.1% (2003–2007) to 0.5% (2008–2012).<br>Congenital abnormalities in 2.9% of live born infants.<br>Earlier initiation of LPV/r and higher maternal CD4+ counts associated with lower MTCT.
Antiretroviral therapy in pregnancy: Balancing the risk of preterm delivery with prevention of mother-to-child HIV transmission	Townsend	2010	United Kingdom Ireland	To evaluate risk–benefit of treatment regimens.	Simulation study; outcomes included risk–benefit of ART.	4,319 births from WLHIV	HAART was associated with a more than sevenfold reduction in MTCT compared with zidovudine monotherapy, but with a 1.4-fold increased odds of preterm delivery and twofold increased odds of severe preterm delivery (<32 weeks).<br>The incremental risk–benefit ratio for HAART in pregnancy compared with monotherapy was 0.63 additional preterm births and 0.23 severe preterm births for each infection prevented.<br>It is estimated that for every 100 HIV transmissions prevented through the use of HAART (rather than monotherapy), 63 additional preterm deliveries would occur, including 23 at <32 weeks gestation.
Pregnancies in older women living with HIV in the UK and Ireland	Townsend	2017	United Kingdom Ireland	To compare outcomes by maternal age.	Population surveillance study; outcomes included maternal age and outcomes.	10 997 WLHIV	15 501 pregnancies<br>The proportion in older women (≥40 years) increased from 2.1% (73 of 3419) in 2000–2004 to 8.9% (510 of 5748) in 2010–2014 (P < 0.001).<br>Compared with pregnancies in younger women, those in older women were more likely to result in multiple birth, stillbirth or an infant with a chromosomal abnormality.<br>However, there was no increased risk of preterm delivery, low birth weight or MTCT among older mothers.

The Experiences and Coping Strategies of United Kingdom-Based African Women Following an HIV Diagnosis During Pregnancy	Treisma n	2014	United Kingdom	To explore experiences following HIV diagnosis in pregnancy.	Qualitative study; outcomes included psychosocial experiences of diagnosis.	12 WLHIV	Themes that emerged included: (a) HIV being part of one's wider tapestry; (b) community and systemic influences and responses to HIV; (c) experiencing a different story of HIV; and (d) the mother–child relationship.<br>Notably, the aspect of HIV that women reported finding most distressing was their inability to breastfeed, which appeared central to their cultural identity as mothers.
Routine HIV counseling and testing during antenatal care in Ukraine: A qualitative study of the experiences and perspectives of pregnant women and antenatal care providers	Tripathi	2013	Ukraine	To assess perceptions of antenatal HIV testing.	Qualitative study; outcomes included HIV testing experiences.	60 pregnant women (15 HIV-positive, 45 HIV-negative) and 25 healthcare providers (15 urban, 10 semi-urban).	Consent: Women described wide variation in informed consent procedures, while providers described these as they are supposed to be. This suggests need for improved training in patient–provider communication.<br>Counseling: Women stated that they had few questions about HIV, while providers described hearing many questions and anxieties about HIV and testing, particularly related to confidentiality and abortion.<br>Post-test counseling may be inadequate given women's fears and concerns.<br>As positive results were commonly met with shock, it may be appropriate to schedule additional counseling at a later time.<br>Providers frequently felt unequipped to provide counseling to HIV-positive women<br>Confidentiality: Clinic procedures may passively jeopardize confidentiality, e.g., by requiring only HIV-positive women to visit certain offices.<br>More semi-urban HIV-positive women reported compromised confidentiality, and it may be important to target semi-urban sites for confidentiality training.
Impact of maternal HIV status on family constructions and the infant's relational environment during the perinatal period.	Trocmé	2013	France	To evaluate impact of maternal HIV on family environment.	Observational study; outcomes included maternal and infant outcomes postpartum.	60 WLHIV and their infants	Half of the 60 mothers did not live with the infant's father<br>56% of multiparous mothers were separated from their previous children.<br>65 % of the fathers were informed of the mother's HIV-positive status, although 90% of fathers who lived with the mothers were informed.<br>During pregnancy, 80% of mothers reported psychological stress<br>After delivery, 72% of mothers suffered from not being allowed to breastfeed their infants<br>43.5% expressed a fear of transmitting the infection to the child, and 40% avoided contacts with the infant.<br>The impact of the mother's psychologicalstress and anxiety related to the risk of HIV transmission through breastfeeding and casual contacts were already noticeable in the first mother–child interrelations.<br>85 % of women were from Sub-Saharan origin<br>Although the risk of MTC transmission in now very small, psychological troubles related to maternal HIV status may negatively affect the

							children’s well-being and behavior, psychological support should be provided for mothers and children as part of comprehensive services.
Maternal Antiretroviral Use and the Risk of Prematurity and Low Birth Weight in Perinatally HIV-Exposed Children—7 Years’ Experience in Two Romanian Centers	Tudor	2026	Romania	To assess associations between HIV, treatment exposure, and birth outcomes.	Retrospective cohort (Romania); outcomes included maternal and infant characteristics.	313 WLHIV and 352 children	Mean maternal age at delivery was 23.1 years, and the mean birth weight of newborns was 2,726 g. Among the children, 191 (54.2%) were male, and the rate of HIV transmission was 13.9%. The prematurity rate was 21.5%, while 25.6% of newborns had low birth weight.<br>Preterm birth was associated with high maternal HIV RNA levels in the third trimester, HIV-positive status in infants, and vaginal delivery. Low birth weight was associated with the absence of antiretroviral treatment during pregnancy and HIV-positive status in infants.<br>No association was found between prematurity or low birth weight in full-term newborns and exposure to any antiretroviral class, specific antiretroviral drugs, number of maternal treatment regimens, duration of antiretroviral therapy prior to conception, or maternal exposure during puberty.<br>In conclusion, preterm birth was significantly associated with vertical HIV transmission and high maternal viral replication during the last trimester of pregnancy. Low birth weight in full-term infants was significantly associated with lack of in utero exposure to antiretroviral therapy.
Comparison of in vitro fertilization cycles in couples with human immunodeficiency virus type 1 infection versus noninfected couples through a retrospective matched case-control study	Vianna	2021	France	To compare assisted reproduction outcomes.	Case–control IVF study; outcomes included live birth rates.	179 IVF cycles in couples infected with HIV-1 and 179 IVF cycles in control couples.	The first comparison between HIV and non-HIV couples showed poorer outcomes in the HIV group (higher administered gonadotropin doses and longer stimulation periods, lower cumulative pregnancy and live birth rates, among other things). A subgroup analysis was performed in addition. No differences were found in the “men HIV” group compared with the controls. In contrast, poorer outcomes in the “women HIV” and “women and men HIV” groups were shown in terms of administered doses, duration of stimulation, and number of oocytes retrieved. For the “women HIV” group, lower cumulative clinical pregnancy and live birth rates were found.<br>The data suggested that couples with HIV-positive women have poorer medically assisted procreation outcomes than couples with non-HIV-infected women.

Prevention of mother-to-child transmission of HIV in Denmark, 1994-2008	von Linstow	2010	Denmark	To describe national trends in pregnancy management.	Retrospective cohort (Denmark); outcomes included maternal and infant outcomes.	210 WLHIV	255 pregnancies; 258 live children.<br>Annual HIV pregnancies increased fivefold.<br>Knowledge of HIV status before pregnancy rose from 8% to 80%.<br>ART use increased from 76% to 98%.<br>Vaginal deliveries ranged from 0% to 35%.<br>MTCT decreased from 10.4% to 0.5%.<br>All HIV transmissions occurred in women undiagnosed or untreated during pregnancy.<br>Median gestational age 38 weeks; 17% preterm; median birth weight 3050 g; 13.4% low birth weight.<br>Physical abnormalities in 16.7% of children.<br>Breastfeeding rare (1.6%).<br>Intrapartum and postnatal prophylaxis widely used among treated women.<br>Maternal CD4 counts and viral suppression improved with earlier ART initiation.
Brief Report: HIV-Positive and Breastfeeding in High-Income Settings: 5-Year Experience From a Perinatal Center in Germany	Weiss	2022	Germany	To evaluate transmission risk, viral suppression, and breastfeeding practices.	Retrospective study; outcomes included transmission and breastfeeding.	181 mother–child pairs among WLHIV	No vertical transmission occurred.<br>83.4% of mothers chose formula feeding; 16.6% breastfed.<br>Among breastfeeding mothers, most were in optimal scenarios with suppressed viral load.<br>cART regimens and infant prophylaxis varied.<br>Breastfeeding might be safe under suppressed maternal viral load and close monitoring
Perception of sexuality and fertility in women living with HIV: a questionnaire study from two Nordic countries	Wessman	2015	Denmark<br>Finland	To investigate perceptions of sexuality and fertility.	Questionnaire study; outcomes included fertility, sexuality, and reproductive health.	560 WLHIV	Median age 44 years.<br>The majority were of white European origin, with fully suppressed HIV viral load, CD4 cell count 350 mL, and mild or no symptoms.<br>Among women of African origin (the largest non-European ethnic group), the vast majority were immigrants<br>62% were sexually active, using condoms as their sole form of contraception; 1/3 of sexually inactive women were in steady relationships.<br>80% reported prior pregnancies, and most children were born before HIV diagnosis through predominantly natural conception.<br>25% desired pregnancy, >50% did not, and the remainder either had the desired number of children or did not answer.<br>14% stated that HIV diagnosis ended their wish for children.<br>46 (8%) were sterilized after diagnosis, of whom 14 (30%) regretted the decision.<br>Pregnancy was unsuccessfully attempted in 134 women.<br>21 (5%) reported having HIV-infected children.<br>Among women with previous or current pregnancies, 49 (10%) had an induced abortion after diagnosis, 39 (8%) a miscarriage, and 70 (15%) a

							live birth.<br>Of 474 women reporting pregnancies, 111 (23%) were pregnant at HIV diagnosis; outcomes included 76 (69%) live births, 18 (16%) induced abortions, 7 (6%) miscarriages, 1 (1%) stillbirth, and 9 (8%) no answer.

Supplementary Table S3. Mixed Methods Appraisal Tool Quality Assessment

Y: Yes; N: No; CT: Can't tell

		SCREENING QUESTIONS		QUALITATIVE STUDIES				
First author	Year	S1. Are there clear research questions?	S2. Do the collected data allow to address the research questions?	1.1. Is the qualitative approach appropriate to answer the research question?	1.2. Are the qualitative data collection methods adequate to address the research question?	1.3. Are the findings adequately derived from the data?	1.4. Is the interpretation of results sufficiently substantiated by data?	1.5. Is there coherence between qualitative data sources, collection, analysis and interpretation?
Alvarez-del Arco	2018	CT	CT	Y	Y	Y	Y	Y
Carlsson-Lalloo	2018	Y	Y	Y	Y	Y	Y	Y
Cooper	2024	Y	Y	Y	Y	Y	Y	Y
Huertas-Zurriaga	2025	Y	Y	Y	Y	Y	Y	Y
Kasadha (How women...)	2024	CT	CT	Y	Y	Y	Y	Y
Kasadha (We decided...)	2024	Y	Y	Y	Y	Y	Y	Y
Kelly	2013	Y	Y	Y	Y	Y	Y	Y
Kelly	2011	CT	CT	Y	Y	Y	Y	Y
King	2019	Y	Y	Y	Y	Y	Y	Y
Lingen-Stallard	2016	Y	Y	Y	Y	Y	Y	Y
Moseholm (Experience of…)	2022	Y	Y	Y	Y	Y	Y	Y
Tariq (Breastfeeding)	2016	CT	CT	Y	Y	Y	N	CT
Treisman	2014	Y	Y	Y	Y	Y	Y	Y
Tripathi	2013	Y	Y	Y	Y	Y	Y	Y
Trocmé	2013	CT	CT	Y	Y	CT	Y	CT
				**3. QUANTITATIVE NON-RANDOMIZED STUDIES**				
		S1. Are there clear research questions?	S2. Do the collected data allow to address the research questions?	3.1. Are the participants representative of the target population?	3.2. Are measurements appropriate regarding both the outcome and intervention (or exposure)?	3.3. Are there complete outcome data?	3.4. Are the confounders accounted for in the design and analysis?	3.5. During the study period, is the intervention administered (or exposure occurred) as intended?
Aebi-Popp	2010	Y	Y	Y	Y	Y	Y	Y
Aebi Popp	2013	Y	Y	Y	Y	CT	Y	Y
Aebi-Popp	2016	Y	Y	Y	Y	Y	Y	Y

Aebi-Popp	2018	Y	Y	Y	Y	N	Y	CT
Ahn	2016	Y	Y	CT	CT	CT	Y	Y
Aho	2018	Y	Y	Y	Y	N	N	Y
Ammassari	2013	Y	Y	Y	Y	N	Y	CT
Azria	2010	Y	Y	CT	Y	Y	CT	Y
Bagkeris	2015	Y	Y	Y	Y	N	Y	CT
Bailey	2010	Y	Y	Y	Y	CT	Y	Y
Bailey	2013	Y	Y	Y	Y	CT	Y	Y
Baroncelli	2011	Y	Y	CT	Y	N	Y	Y
Baroncelli	2015	Y	Y	CT	Y	CT	Y	Y
Baza	2019	Y	Y	CT	Y	CT	Y	Y
Brandon	2022	Y	Y	N	Y	Y	N	CT
Byrne	2017	Y	Y	Y	Y	N	Y	Y
Cabalak	2024	Y	Y	CT	Y	CT	N	Y
Cambrea	2022	Y	Y	CT	Y	CT	N	Y
Carey	2018	Y	Y	N	Y	N	N	CT
Del Romero	2016	Y	Y	CT	Y	Y	CT	Y
Dema	2024	Y	Y	Y	Y	CT	Y	Y
Di Biagio	2019	Y	Y	CT	Y	Y	Y	Y
Dorobat	2014	N	CT	CT	Y	CT	CT	Y
European Collaborative Study (Factors associated...)	2010	Y	Y	Y	Y	CT	Y	Y
European Collaborative Study (Mode of delivery…)	2010	Y	Y	CT	Y	N	Y	N
Favarato	2018	Y	Y	Y	Y	CT	Y	Y
Favarato	2019	Y	Y	Y	Y	CT	Y	CT
Floridia	2014	Y	Y	CT	Y	CT	Y	Y
Floridia	2015	Y	Y	Y	Y	CT	Y	Y
Floridia	2017	CT	CT	N	Y	Y	N	CT
Floridia	2019	Y	Y	CT	Y	N	Y	CT
Floridia	2020	Y	Y	Y	Y	CT	Y	Y
Floridia	2021	Y	Y	CT	Y	N	Y	CT
French	2012	Y	Y	Y	Y	CT	Y	Y
French	2017	Y	Y	Y	Y	N	Y	Y
Grignolo	2017	Y	Y	Y	Y	N	Y	CT
Hernando	2017	Y	Y	N	Y	N	Y	CT
Hofacker	2024	Y	Y	Y	Y	CT	Y	CT
Huntington	2013	Y	Y	CT	Y	CT	Y	CT

Huntington	2015	Y	Y	CT	Y	Y	Y	Y
Hurt	2026	Y	Y	Y	Y	CT	CT	Y
Illan-Ramos	2024	Y	Y	Y	Y	Y	CT	CT
Inkaya	2020	Y	Y	CT	Y	CT	N	CT
Izzo	2011	Y	Y	CT	Y	CT	Y	Y
Jasseron	2013	Y	Y	Y	Y	N	Y	CT
King	2021	Y	Y	N	Y	CT	Y	CT
Liuzzi	2013	Y	Y	CT	Y	N	Y	CT
Lopez	2022	Y	Y	Y	Y	N	Y	N
Lumbreras Areta	2024	Y	Y	CT	Y	CT	Y	CT
Montgomery-Taylor	2015	Y	Y	CT	CT	CT	N	Y
Moseholm	2025	Y	Y	Y	Y	Y	Y	Y
Mutru	2023	Y	Y	Y	Y	Y	CT	Y
Nöstlinger	2013	Y	Y	N	Y	N	Y	CT
Nyatsanza	2021	Y	Y	N	Y	CT	N	Y
Okhai	2021	Y	Y	CT	Y	Y	Y	Y
Okhai	2025	Y	Y	Y	Y	N	Y	Y
Ørbæk	2017	Y	Y	Y	Y	Y	Y	Y
Paioni	2023	Y	Y	CT	Y	N	Y	CT
Pammi	2015	Y	Y	Y	Y	CT	Y	CT
Peters	2017	Y	Y	Y	Y	Y	N	CT
Peters	2018	Y	Y	Y	Y	N	N	Y
Pilecco	2024	Y	Y	N	Y	N	Y	CT
Pontiki	2022	Y	Y	N	Y	Y	Y	CT
Prieto	2017	Y	Y	CT	Y	Y	CT	CT
Rachas	2013	Y	Y	Y	Y	Y	Y	Y
Reitter	2014	Y	Y	CT	Y	N	CT	CT
Rudin	2011	Y	Y	Y	Y	CT	Y	Y
Ruiz-Alguero	2023	Y	Y	CT	Y	N	N	CT
Saxton	2010	Y	Y	CT	CT	CT	Y	Y
Sibiude	2012	Y	Y	Y	Y	CT	Y	Y
Sibiude	2023	Y	Y	Y	Y	N	N	CT
Suarez Garcia (Pregnancy in…)	2025	Y	Y	Y	Y	N	N	CT
Suarez Garcia (Diagnosis of…)	2025	Y	Y	Y	Y	Y	Y	Y
Tariq	2016	Y	Y	CT	Y	CT	Y	Y
Thorne	2012	Y	Y	CT	Y	CT	Y	Y
Tibaldi	2019	Y	Y	Y	Y	Y	N	N
Tookey	2016	Y	Y	Y	Y	Y	CT	Y
Townsend	2010	Y	Y	CT	Y	CT	Y	Y

Townsend	2017	Y	Y	Y	Y	CT	Y	Y
Tudor	2026	Y	Y	CT	Y	N	N	CT
Vianna	2021	Y	Y	CT	Y	Y	Y	Y
von Linstow	2010	Y	Y	Y	Y	CT	N	Y
Weiss	2022	Y	Y	CT	Y	CT	N	Y
Wessman	2015	Y	Y	CT	Y	N	Y	CT
				**QUANTITATIVE DESCRIPTIVE STUDIES**				
		S1. Are there clear research questions?	S2. Do the collected data allow to address the research questions?	4.1. Is the sampling strategy relevant to address the research question?	4.2. Is the sample representative of the target population?	4.3. Are the measurements appropriate?	4.4. Is the risk of nonresponse bias low?	4.5. Is the statistical analysis appropriate to answer the research question?
Bailey	2014	Y	Y	Y	Y	Y	CT	Y
Bailey	2016	Y	Y	Y	Y	Y	Y	Y
Crisinel	2023	Y	Y	Y	CT	Y	N	Y
Haberl	2021	Y	Y	Y	N	Y	N	Y
Loftus	2016	CT	CT	Y	CT	Y	CT	Y
Raffe	2017	Y	Y	Y	Y	CT	Y	CT
				**MIXED METHODS STUDIES**				
		S1. Are there clear research questions?	S2. Do the collected data allow to address the research questions?	5.1. Is there an adequate rationale for using a mixed methods design to address the research question?	5.2. Are the different components of the study effectively integrated to answer the research question?	5.3. Are the outputs of the integration of qualitative and quantitative components adequately interpreted?	5.4. Are divergences and inconsistencies between quantitative and qualitative results adequately addressed?	5.5. Do the different components of the study adhere to the quality criteria of each tradition of the methods involved?
Hernando	2014	Y	Y	N	CT	CT	CT	CT
Moseholm	2022	Y	Y	N	N	N	Y	Y
Moseholm (Infant feeding knowledge...)	2024	Y	Y	Y	Y	Y	Y	Y
Moseholm (Perception and emotional...)	2024	Y	Y	CT	Y	Y	Y	Y

Hong, Q. N., Fàbregues, S., Bartlett, G., Boardman, F., Cargo, M., Dagenais, P., Gagnon, M.-P., Griffiths, F., Nicolau, B., O'Cathain, A., Rousseau, M.-C., Vedel, I., & Pluye, P. 2018. The Mixed Methods Appraisal Tool (MMAT) version 2018 for information professionals and researchers. Education for Information, 34(4), 285-291.

Supplementary Table S4. MMAT Quality Assessment by Study Design and Criterion

**Qualitative studies (n = 15)**				
S1	Clear research questions	10/15 (67%)	5/15 (33%)	0/15 (0%)
S2	Data allow addressing research questions	10/15 (67%)	5/15 (33%)	0/15 (0%)
1.1	Appropriate qualitative approach	15/15 (100%)	0/15 (0%)	0/15 (0%)
1.2	Adequate data collection methods	15/15 (100%)	0/15 (0%)	0/15 (0%)
1.3	Findings adequately derived from data	14/15 (93%)	1/15 (7%)	0/15 (0%)
1.4	Interpretation substantiated by data	14/15 (93%)	0/15 (0%)	1/15 (7%)
1.5	Coherence across data sources, collection, analysis, and interpretation	13/15 (87%)	2/15 (13%)	0/15 (0%)
**Quantitative non-randomized studies (n = 82)**				
S1	Clear research questions	79/82 (96%)	2/82 (2%)	1/82 (1%)
S2	Data allow addressing research questions	79/82 (96%)	3/82 (4%)	0/82 (0%)
3.1	Participants representative of target population	40/82 (49%)	31/82 (38%)	11/82 (13%)
3.2	Appropriate measurements (outcome and exposure)	80/82 (98%)	2/82 (2%)	0/82 (0%)
3.3	Complete outcome data	16/82 (20%)	40/82 (49%)	26/82 (32%)
3.4	Confounders accounted for in design and analysis	45/82 (55%)	15/82 (18%)	22/82 (27%)
3.5	Intervention/exposure administered as intended	50/82 (61%)	31/82 (38%)	1/82 (1%)
**Quantitative descriptive studies (n = 6)**				
S1	Clear research questions	5/6 (83%)	1/6 (17%)	0/6 (0%)
S2	Data allow addressing research questions	5/6 (83%)	1/6 (17%)	0/6 (0%)
4.1	Relevant sampling strategy	6/6 (100%)	0/6 (0%)	0/6 (0%)
4.2	Sample representative of target population	3/6 (50%)	2/6 (33%)	1/6 (17%)
4.3	Appropriate measurements	5/6 (83%)	1/6 (17%)	0/6 (0%)
4.4	Low risk of nonresponse bias	2/6 (33%)	2/6 (33%)	2/6 (33%)
4.5	Appropriate statistical analysis	5/6 (83%)	1/6 (17%)	0/6 (0%)
**Mixed methods studies (n = 4)**				
S1	Clear research questions	4/4 (100%)	0/4 (0%)	0/4 (0%)
S2	Data allow addressing research questions	4/4 (100%)	0/4 (0%)	0/4 (0%)
5.1	Adequate rationale for mixed methods design	1/4 (25%)	1/4 (25%)	2/4 (50%)
5.2	Effective integration of study components	2/4 (50%)	1/4 (25%)	1/4 (25%)
5.3	Adequate interpretation of integrated outputs	2/4 (50%)	1/4 (25%)	1/4 (25%)
5.4	Divergences and inconsistencies addressed	3/4 (75%)	1/4 (25%)	0/4 (0%)
5.5	Adherence to quality criteria of each method	3/4 (75%)	1/4 (25%)	0/4 (0%)

Supplementary Table S5. Preferred Reporting Items for Systematic reviews and Meta-Analyses extension for Scoping Reviews (PRISMA-ScR) Checklist

SECTION	ITEM	PRISMA-ScR CHECKLIST ITEM	REPORTED ON PAGE #
**TITLE**			
Title	1	Identify the report as a scoping review.	1
**ABSTRACT**			
Structured summary	2	Provide a structured summary that includes (as applicable): background, objectives, eligibility criteria, sources of evidence, charting methods, results, and conclusions that relate to the review questions and objectives.	1
**INTRODUCTION**			
Rationale	3	Describe the rationale for the review in the context of what is already known. Explain why the review questions/objectives lend themselves to a scoping review approach.	1-4
Objectives	4	Provide an explicit statement of the questions and objectives being addressed with reference to their key elements (e.g., population or participants, concepts, and context) or other relevant key elements used to conceptualize the review questions and/or objectives.	4
**METHODS**			
Protocol and registration	5	Indicate whether a review protocol exists; state if and where it can be accessed (e.g., a Web address); and if available, provide registration information, including the registration number.	3
Eligibility criteria	6	Specify characteristics of the sources of evidence used as eligibility criteria (e.g., years considered, language, and publication status), and provide a rationale.	4-5
Information sources*	7	Describe all information sources in the search (e.g., databases with dates of coverage and contact with authors to identify additional sources), as well as the date the most recent search was executed.	4-5
Search	8	Present the full electronic search strategy for at least 1 database, including any limits used, such that it could be repeated.	Supplementary material 1
Selection of sources of evidence†	9	State the process for selecting sources of evidence (i.e., screening and eligibility) included in the scoping review.	4-5, 7
Data charting process‡	10	Describe the methods of charting data from the included sources of evidence (e.g., calibrated forms or forms that have been tested by the team before their use, and whether data charting was done independently or in duplicate) and any processes for obtaining and confirming data from investigators.	5

SECTION	ITEM	PRISMA-ScR CHECKLIST ITEM	REPORTED ON PAGE #
Data items	11	List and define all variables for which data were sought and any assumptions and simplifications made.	5
Critical appraisal of individual sources of evidence§	12	If done, provide a rationale for conducting a critical appraisal of included sources of evidence; describe the methods used and how this information was used in any data synthesis (if appropriate).	6
Synthesis of results	13	Describe the methods of handling and summarizing the data that were charted.	6
**RESULTS**			
Selection of sources of evidence	14	Give numbers of sources of evidence screened, assessed for eligibility, and included in the review, with reasons for exclusions at each stage, ideally using a flow diagram.	7
Characteristics of sources of evidence	15	For each source of evidence, present characteristics for which data were charted and provide the citations.	Supplementary material 2
Critical appraisal within sources of evidence	16	If done, present data on critical appraisal of included sources of evidence (see item 12).	Supplementary material 3
Results of individual sources of evidence	17	For each included source of evidence, present the relevant data that were charted that relate to the review questions and objectives.	Supplementary material 2
Synthesis of results	18	Summarize and/or present the charting results as they relate to the review questions and objectives.	9
**DISCUSSION**			
Summary of evidence	19	Summarize the main results (including an overview of concepts, themes, and types of evidence available), link to the review questions and objectives, and consider the relevance to key groups.	7-17
Limitations	20	Discuss the limitations of the scoping review process.	21-22
Conclusions	21	Provide a general interpretation of the results with respect to the review questions and objectives, as well as potential implications and/or next steps.	22-23
**FUNDING**			
Funding	22	Describe sources of funding for the included sources of evidence, as well as sources of funding for the scoping review. Describe the role of the funders of the scoping review.	23

From: Tricco AC, Lillie E, Zarin W, O'Brien KK, Colquhoun H, Levac D, et al. PRISMA Extension for Scoping Reviews (PRISMAScR): Checklist and Explanation. Ann Intern Med. 2018;169:467–473. doi: 10.7326/M18-0850
